# Adaptive filter parameter reconstruction technology for rocket inertial navigation/satellite integrated navigation system

**DOI:** 10.7717/peerj-cs.3040

**Published:** 2025-07-23

**Authors:** Zhijie Yang, Guoguang Chen, Mingli Niu, Xiaolong Yan, Xiaoli Tian, Guocui Zhang

**Affiliations:** 1 College of Mechanical and Electrical Engineering, North University of China, Taiyuan, China; 2 Mechanical Engineering College, Taiyuan Railway Machinery School, Taiyuan, China; 3 Jinxi Industrial Group Company Ltd, Taiyuan, China

**Keywords:** Guided rockets, MEMS-SINS/GNSS, Integrated navigation, Extended Kalman filter, Adaptive parameters

## Abstract

The integration of micro-electro-mechanical systems (MEMS) strapdown inertial navigation systems (SINS) with global navigation satellite systems (GNSS) has emerged as a significant area of research due to its compact size, affordability, and high precision. In the context of guided rocket-borne MEMS-SINS/GNSS integrated navigation systems, the performance of navigation is characterized by the need for high overload, accuracy, and real-time capability. A variety of enhanced algorithms based on Kalman filtering are currently employed as integrated filtering methods, which comprehensively address deviations in the system model to improve navigation performance. The noise characteristics of MEMS inertial guidance devices change dramatically under long-term storage conditions, while the dynamic flight environment of rockets and the high real-time requirements of navigation solving make the design of on-board combined navigation filters challenging. To address this issue, this article introduces the Adaptive Reconfigurable Extended Kalman Filter (AREKF) method. Initially, a precise system state model is developed to reflect the unique characteristics of the rocket flight environment, facilitating rapid convergence of the filtering process. Subsequently, during the rocket alignment process, a real-time reconstruction of filter parameters is implemented to enable adaptive and precise modeling of navigation parameters. This strategy ensures lower computational costs during rocket flight, enhances the accuracy of the navigation system, and produces real-time navigation outputs that exhibit high overload and precision. The results from the Six-Degree (6D) Model simulation and car-mounted experiments demonstrate that, compared to the traditional Extended Kalman Filter (EKF) algorithm and existing improved algorithms, the AREKF method significantly enhances the real-time navigation accuracy of rockets under high overload conditions.

## Introduction

The navigation system plays a crucial role in guiding rockets and is essential for achieving precise strikes. The design of the rocket navigation system must meet the demands of high overload, exceptional accuracy, and strict real-time performance (Zhao et al., 2022). To date, advancements in the performance of rocket navigation systems have concentrated on two main aspects: the composition of the integrated navigation system (Zhang et al., 2024a, 2024b) and the design of the navigation information fusion filter algorithm (Liu, Wang & Abel, 2023; Xiao et al., 2021). The micro-electro-mechanical systems (MEMS) strapdown inertial navigation system (MEMS-SINS) is distinguished by its all-weather capabilities and full autonomy in navigation. It also features strong dynamic characteristics, a compact size, and low cost, making it a suitable navigation system for rockets (Abdolkarimi & Mosavi, 2020; Zhao et al., 2023). However, MEMS inertial navigation devices inherently exhibit drift characteristics due to their processing and structural design, resulting in navigation deviations that accumulate over time (Hu et al., 2020b; Xiong et al., 2019). Consequently, MEMS-SINS must be supplemented and corrected by other navigation systems to improve navigation accuracy (Shen et al., 2022; Vinoj & Lalu, 2024). Currently, the global navigation satellite system (GNSS) provides highly accurate positional and speed information with no cumulative error. However, its data update frequency is relatively low, and it is susceptible to environmental influences (Chen, Liu & Fang, 2022; Wang & Zhang, 2024). Consequently, GNSS cannot function independently as a navigation system for rockets. Instead, a combined MEMS-SINS/GNSS navigation system, which integrates satellite navigation and inertial navigation systems, offers complementary advantages (Liu et al., 2023; Tang et al., 2024). This integration significantly enhances the performance of rocket navigation systems. For these reasons, the MEMS-SINS/GNSS system has become a widely adopted navigation scheme for guided rockets.

In the strapdown navigation system, attitude determination is a critical component of navigation information calculation. The integration of multiple navigation systems can leverage complementary advantages, facilitating precise determination of attitude information. Hu et al. (2016b) enhanced computational efficiency and navigation robustness to some extent by adopting a distributed scheme and parallel data processing; however, this improvement came at the expense of navigation solution accuracy. Conversely, Gao et al. (2022a) achieved accurate navigation attitude information through the implementation of a novel distributed technology for multi-sensor navigation information fusion. Furthermore, Hu et al. (2023) introduced a scalar factor adjustment gain *via* Mahalanobis distance hypothesis testing to mitigate the impact of abnormal measurements, although this approach resulted in reduced computational efficiency. Other scholars have improved navigation performance and computational efficiency by employing matrix adaptive random weighted data fusion (Gao, Zhong & Li, 2011a; Gao, Zhong & Shirinzadeh, 2010; Hu et al., 2016a), yet the selection of weighting coefficients presents challenges for the practical engineering implementation of this method (Gao et al., 2020). Gao et al. (2009) enhanced system stability and accuracy under the abnormal conditions of a single navigation system by utilizing local decentralized fusion and global optimal fusion techniques. Jia et al. (2019) and Gao et al. (2018c) further improved system robustness and accuracy by adjusting equivalent weight matrices and adaptive factors. Gao et al. (2019b) achieved precise navigation system solutions under strong wind conditions by incorporating a wind speed model, although this method necessitated substantial data support. Gao et al. (2018b) enhanced the robustness of local state estimation by employing an adaptive fading unscented Kalman filter based on Mahalanobis distance, achieving global optimization through the unscented transformation multi-sensor data fusion method. This significantly improved navigation performance, although it introduced higher computational complexity and challenges in real-time solution noise. Meanwhile, Gao et al. (2021d) implemented fault detection in multi-sensor systems using dual-channel sequential probability ratio testing, effectively isolating filtered measurement data. While multi-sensor fusion technology can enhance the robustness and accuracy of navigation systems (Gao et al., 2023c), it also increases the system’s volume and reduces the computational efficiency of filtering algorithms, presenting certain challenges in the application to cost-effective rocket projectile navigation systems.

In-depth research on integrated filtering technology is a key factor in enhancing navigation performance. Gao et al. (2023b) constrained the maximum likelihood estimation through the chi-square test and the improved sequential probability ratio test, effectively addressing the impact of measurement outliers on filtering performance. Hu, Gao & Zhong (2015) adopted a tightly coupled filtering design by retaining the second-order Taylor terms, which reduced modeling errors caused by linearization and enhanced integrated navigation performance. Furthermore, Hu et al. (2018) improved the accuracy of the navigation system by designing a hypothesis function to assess motion model errors and introducing a suboptimal attenuation factor to refine the prediction covariance matrix, although this increased the design complexity. Meng et al. (2016) utilized the innovation sequence and the residual sequence to estimate the noise covariance matrix, thereby minimizing the discrepancy between it and the theoretical value, which improved navigation performance. It is well known that closed-loop design can enhance control accuracy, Gao et al. (2023a) incorporated the covariance matrix back into the filtering process, established a feedback control system based on the maximum likelihood principle, and designed a reasonable proportional coefficient to improve navigation system performance. However, the effectiveness of this scheme is contingent upon the selection of the proportional coefficient, which adds complexity to the filtering design. Gao et al. (2021b, 2019a, 2022b) extended the system state by incorporating dynamic noise and utilized the Mahalanobis distance to detect uncertainties in the dynamic model. They also calculated attenuation factors to enhance the convergence speed under uncertain models. Gao, Zhong & Li (2011b) designed an adaptive adjustment factor to regulate the system state covariance matrix, which improved navigation accuracy for uncertain systems. However, as the dimensionality of the system state increases, the computational complexity escalates, presenting challenges for dynamic real-time systems.

The CKF method can suppress the nonlinear errors of the integrated navigation system (Zong et al., 2019). Gao et al. (2021c) established a motion error identification and prediction model based on the Mahalanobis distance theory to improve state estimation accuracy. However, the error identification relies on the setting of the Mahalanobis distance threshold, which limits the system stability. Hu et al. (2024) designed a fuzzy inference system to adjust the measurement noise covariance, enhancing the filter convergence speed, but the system’s effectiveness is overly dependent on the design of fuzzy rules. Zhang et al. (2019) integrated joint probabilistic data association into the CKF and employed an improved Sage-Husa maximum *a posteriori* method to enhance state estimation accuracy. Gao et al. (2019c) proposed a stochastic weighting technique under dynamic measurement sampling based on the CKF, improving estimation accuracy and system adaptability. However, while these algorithms enhance accuracy, they also increase computational complexity, making it challenging to meet the real-time requirements of rocket navigation systems.

The CKF methods exhibit substantial computational demands, posing challenges to the real-time requirements of rocket navigation systems. In contrast, the EKF method employs the first-order Taylor expansion to address nonlinear system processing, resulting in enhanced computational speed and real-time performance (Li et al., 2024). This makes the EKF method more suitable for implementation in the combined navigation system of rockets. Li & Chang (2020) conducted a reconstruction of the attitude error model using the multiplicative extended Kalman filter (MKEKF), which enhances navigation performance. However, this approach exhibits poor robustness against uncertain model errors. Vinoj & Lalu (2024) commenced with the classical EKF algorithm, enhancing integrated navigation accuracy in scenarios involving loss of lock satellites by expanding the deformed filtering state array, Nevertheless, this method is characterized by slow convergence during filtering. Ibrahim et al. (2023) proposed an invariant extended Kalman filter (IEKF) based loose coupling scheme to enhance navigation and positioning accuracy in scenarios involving GNSS rejection. Similarly, Taghizadeh et al. (2022) developed a new factor graph optimization approach based on the EKF filter, which integrates data from both the magnetometer and the barometer. This method improves navigation performance during GNSS outages without significantly increasing the system’s computation time (Taghizadeh et al., 2022); however, it does not enhance the overall performance of combined navigation. Li, Zhang & Wang (2021) proposed an auto regressive integrated moving average (ARIMA) auxiliary model based on time sequence to enhance the navigation performance of the Extended Kalman Filter. However, the complexity of the algorithm design poses challenges for achieving highly dynamic real-time output. Zhao et al. (2022) introduced a Maximum Visoria Criterion Extended Kalman Filter (MVC-EKF) algorithm that leverages the concepts of MVC and M-estimation to maintain a low computational cost while improving the suppression of non-Gaussian noise. Nonetheless, as the number of filtering states increases, the algorithm’s enhancement effect becomes limited. Luo, Guo & Chen (2023) propose a filter and a piecewise smoother based on the matrix Lie group of double direct isometries (SE2(3)) to enhance real-time performance and convergence. However, the algorithm primarily focuses on long-term filtering effects, which limits its effectiveness for ground-to-ground guided rockets with flight times on the order of minutes.

The estimation of system noise in inertial navigation devices is a crucial technology for enhancing the accuracy of navigation systems. Gao et al. (2025, 2024) proposed methods such as the H∞ filter and random weighting estimation technique for system noise estimation, which do not require statistical noise characteristics. These methods exhibit strong anti-interference capabilities, high estimation accuracy, and simple computation. However, they primarily target linear systems, necessitating further research into their applicability to nonlinear systems. Gao et al. (2017) integrated the maximum posterior and random weighting techniques with filtering methods to investigate the characteristics of nonlinear systems, thereby improving the system’s online estimation accuracy and computational efficiency. Nonetheless, the determination of weighting factors heavily relies on engineering experience, which can adversely affect the filter’s performance. Hu et al. (2020b) employed the maximum likelihood principle to estimate process noise covariance, thereby enhancing the robustness of the unscented Kalman filter against noise uncertainty. Gao et al. (2021a) proposed a measurement noise covariance estimation method based on the maximum likelihood and sequential quadratic programming techniques using the CKF, which improves navigation accuracy while also enhancing robustness. However, this method exhibits high computational complexity, making it challenging to optimize both navigation performance and computational efficiency under dynamic conditions. Gao, Hu & Zhong (2015) combined windowing and random weighting to address the statistical requirements of noise characteristics in the unscented Kalman filter, significantly enhancing filtering accuracy and robustness under dynamic real-time conditions. However, this method struggles with estimating complex noise characteristics. To mitigate this issue, Gao et al. (2018a) integrated moving horizon estimation and expectation maximization techniques with machine learning to improve the handling of complex noise characteristics and enhance filtering performance. Zhang et al. (2019) adopted the joint probabilistic data association principle and combined it with a distributed noise statistical estimator to address the problem of inaccurate measurement noise. However, this approach entails high computational complexity, posing challenges for real-time applications.

Based on the aforementioned research findings, and considering the high overload, precision, and real-time characteristics of the rocket navigation system, this article proposes an adaptive reconfiguration extended Kalman filter (AREKF) technique that utilizes a MEMS-SINS/GNSS loosely-coupled scheme. This study implements an adaptive real-time estimation of the extended Kalman filter (EKF) parameters during the pre-rocket alignment phase to facilitate the reconfiguration of the integrated navigation filter parameters for rocket flight. The following advantages are provided:

(a) In this article, we employ the EKF method with a 15th-order error state matrix. This approach offers high reliability and computational efficiency compared to other schemes such as the cubature Kalman filter (CKF) and the unscented Kalman filter (UKF). Additionally, the second-order nonlinear term in the Taylor expansion is linearized with a single error, enhancing the modeling accuracy of the navigation system.
(b) In this article, we present a method for estimating the noise characteristics of pre-launch inertial equipment. By reconstructing the filter parameters, we mitigate the impact of noise characteristic changes that occur during the prolonged storage of rockets. This approach enables the precise design of EKF parameters at a significantly reduced cost, offering higher computational efficiency compared to existing inertial equipment noise estimation schemes. Consequently, our method achieves high real-time processing of the rocket’s navigation information.

This article presents an adaptive parameter reconstruction method using the EKF to address the significant changes in noise characteristics that occur during the long-term storage of MEMS inertial equipment on rockets. It demonstrates that the original filter parameter design is inadequate for the current mission requirements of the system. Additionally, the precise reconstruction of navigation filter parameters enhances the adaptability to the dynamic flight environment of the rocket and improves the accuracy of the navigation solver. Furthermore, a corresponding simulation and verification platform is established for this filter design method, and the experimental results confirm the superiority of the proposed algorithm.

The remainder of this article is organized as follows: “Principle of AREKF Technology” outlines the design methodology of the AREKF for the integrated navigation system of rockets utilizing MEMS-SINS/GNSS. “Digital Simulation” presents a numerical simulation of the MEMS-SINS/GNSS integrated navigation system, based on the six-degree (6D) trajectory of a rocket, while comparing the navigation performance of various methods. “Car-Mounted Experiments for SINS/GNSS Integration Navigation” describes the car-mounted experiments of the integrated navigation system and evaluates the performance of the proposed method. Finally, “Conclusion” summarizes the research results.

## Principle of AREKF technology

### SINS/GNSS integrated navigation EKF technical principle

The SINS equations are the core of the inertial navigation/satellite EKF algorithm, which realizes the navigation solution of the attitude, velocity and position information under pure inertial conditions, including the attitude update equations, velocity update equations and attitude update equations of strapdown inertial navigation. Equation (1) shows the attitude update equation (Ibrahim et al., 2023; Zhao et al., 2023).

(1) $$\left\{ \matrix{ { \dot {q} }= {1 \over 2} {q }\otimes \left[ {\matrix{ 0 \cr {\bf{\omega }} \cr } } \right] \hfill \cr C_b^n = \left[ {\matrix{ {q_x^2 + q_s^2 - q_y^2 - q_z^2} & {2({q_x}{q_y} - {q_z}{q_s})} & {2({q_y}{q_z} + {q_y}{q_s})} \cr {2({q_x}{q_y} + {q_z}{q_s})} & {q_y^2 + q_s^2 - q_x^2 - q_z^2} & {2({q_y}{q_z} - {q_x}{q_s})} \cr {2({q_y}{q_z} - {q_y}{q_s})} & {2({q_y}{q_z} + {q_x}{q_s})} & {q_z^2 + q_s^2 - q_x^2 - q_y^2} \cr } } \right] . \hfill \cr} \right. $$

${\bf \omega }$ is the angular velocity of rocket rotation, ${{q}} = [{q_s},{q_x},{q_y},{q_z}]$ are conversion quaternions for different coordinate systems, the operator $\otimes$ is the quaternion multiplication notation. For the detailed rules of the quaternion, please refer to article 36 (Santamaria-Navarro et al., 2018), $C_b^n$ is the transformation matrix from the bomb system to the navigation system;

Equations (2) and (3) show the velocity update equation and the position update equation (Ibrahim et al., 2023).

(2) $${ \dot { v} }= { f}_{sf}^b - (2*{{\boldsymbol  \omega }_{ie}} + {{\boldsymbol  \omega}_{en}}) \times { v + g}$$

(3) $$\dot p = \left[ \matrix{{\dot L} \hfill \cr {\dot \lambda } \hfill \cr {\dot h} \hfill} \right] = \left[ \matrix{{v_N}/(h + {R_M}) \hfill \cr {v_E}*\sec L/(h + {R_M}) \hfill \cr {v_U} \hfill} \right].$$

${v}$ is speed information; ${ f}_{sf}^b$ is accelerometer output information; ${{\bf \omega }_{ie}}$ is angular velocity of the Earth’s rotation; ${{\bf \omega }_{en}}$ is the rotational angular velocity of the navigation system relative to the Earth’s coordinate system; ${g}$ is gravity acceleration information; t is time; $L$ is geographic latitude; $\lambda$ is geographic longitude; $h$ is geographic altitude, ${R_M}$ and ${R_N}$ are the median radius and normal radius (Hu et al., 2020a).

The general form of Taylor’s expansion states that for any state quantity, the specific formula for the Taylor expansion within a given time interval is presented in Eq. (4).

(4) $$x(t + \Delta t) = x(t) + \dot x(t)\Delta t + \displaystyle{1 \over 2}\ddot x(t)\Delta{t^2} + \cdots + \displaystyle{1 \over {n!}}{x^{(n)}}(t)\Delta{t^n} + o(\Delta{t^{n + 1}}).$$

The combined SINS/GNSS navigation aims to achieve precise solutions for position, velocity, and attitude information at each discrete moment. The exact solutions for the equations of position, velocity, and attitude information, derived using Taylor expansion, are presented in Eq. (5).

(5) $$\left\{ \matrix{{p(t + \Delta t) = p(t) + \dot p(t)\Delta t + \displaystyle{1 \over 2}\ddot p(t)\Delta{t^2} + \cdots + \displaystyle{1 \over {n!}}{p^{(n)}}(t)\Delta{t^n} + o(\Delta{t^{n + 1}})}\hfill \cr {v(t + \Delta t) = v(t) + \dot v(t)\Delta t + \displaystyle{1 \over 2}\ddot v(t)\Delta{t^2} + \cdots + \displaystyle{1 \over {n!}}{v^{(n)}}(t)\Delta{t^n} + o(\Delta{t^{n + 1}})}\hfill \cr {C_b^n(t + \Delta t) = C_b^n(t) + \dot C_b^n(t)\Delta t + \displaystyle{1 \over 2}\ddot C_b^n(t)\Delta{t^2} + \cdots + \displaystyle{1 \over {n!}}C{_b^{n^{(n)}}}(t)\Delta{t^n} + o(\Delta{t^{n + 1}}). }} \right.$$

The inertial navigation differential equations represented by Eqs. (1) to (3) encompass only the first-order term in the Taylor expansion of the navigation information $\dot p,\dot v,\dot C_b^n$. However, in a rocket navigation system characterized by high overload performance, the influence of the second-order term is significant and cannot be disregarded. In the design of the combined SINS/GNSS navigation system, the Taylor expansion is illustrated in Eq. (6), where the error-extended Kalman filtering design is employed, treating the error term of the navigation information as a state variable.

(6) $$\left\{ \matrix{ \Delta p(t + \Delta t) = \Delta p(t) + \Delta \dot p(t)\Delta t + o(\Delta {t^3}) \hfill \cr  \Delta v(t + \Delta t) = \Delta v(t) + \Delta \dot v(t)\Delta t + o(\Delta {t^3}) \hfill \cr  \Delta C_b^n(t + \Delta t) = \Delta C_b^n(t) + \Delta \dot C_b^n(t)\Delta t + o(\Delta {t^3}). \hfill \cr} \right.$$

The second-order term of the navigation Taylor expansion $\ddot x(t)$ in Eq. (4) has been linearized using error linearization $\Delta\dot x(t)$. This process achieves a single linearization of the second-order term, followed by the application of the EKF method to enhance the dynamic solving accuracy of the rocket navigation system.

The inertial navigation error transfer equations are the basis for building the EKF filter equations, the principle of extended Kalman filtering is a one-time linearization of the nonlinear model error; the filtering function of the nonlinear system is realized by omitting the second-order term, and the SINS error transfer equation is established according to the inertial navigation update Eqs. (1)~(3) and (7) shows the state variables in the error transfer equations.

(7) $${{ X}_G} = {[{\delta _L},{\delta _\lambda },{\delta _H},{\delta _{{v_E}}},{\delta _{{v_N}}},{\delta _{{v_U}}},{\phi _E},{\phi _N},{\phi _U},{\varepsilon _x},{\varepsilon _y},{\varepsilon _z},{\nabla _x},{\nabla _y},{\nabla _z}]^T}$$

where ${\delta _L},{\delta _\lambda },{\delta _H}$ represent the deviations in longitude, latitude, and altitude, respectively. Meanwhile, ${\delta _{{v_E}}},{\delta _{{v_N}}},{\delta _{{v_U}}}$ are the deviations in east velocity, north velocity, and up velocity. Additionally, ${\varepsilon _x},{\varepsilon _y},{\varepsilon _z}$ signify the gyro zero bias, while ${\nabla _x},{\nabla _y},{\nabla _z}$ also represent the accelerometer zero bias.

The inertial navigation error transfer equation is shown in Eq. (8) (Hu et al., 2023, 2019).

(8) $${{\dot { X} }_G}(t) = {{ F}_G}(t){{ X}_G}(t) + {{ G}_G}(t){{ W}_G}(t).$$

${{ F}_G}(t)$ is the system state matrix, the parameters of each variable in the matrix are shown in Eq. (9).

(9) $${{ F}_G}(t) = \left[ {\matrix{ {{{{F}}_{{G_{11}}}}} & {{{{F}}_{{G_{12}}}}} & {{0_{3 \times 3}}} & {{0_{3 \times 3}}} & {{0_{3 \times 3}}} \cr {{{{F}}_{{G_{21}}}}} & {{{F}_{{G_{22}}}}} & {{0_{3 \times 3}}} & {{0_{3 \times 3}}} & { - C_b^n} \cr {{{{F}}_{{G_{31}}}}} & {{{{F}}_{{G_{32}}}}} & {{{{F}}_{{G_{33}}}}} & { - C_b^n} & {{0_{3 \times 3}}} \cr {{0_{6 \times 3}}} & {{0_{6 \times 3}}} & {{0_{6 \times 3}}} & {{0_{6 \times 3}}} & {{0_{6 \times 3}}} \cr } } \right].$$

Equation (6) has the following definitions:

${{{F}}_{{G_{11}}}} = \left[ {\matrix{ 0 & 0 & { - \displaystyle{{{v_N}} \over {{{({R_M} + h)}^2}}}} \cr {\displaystyle{{{v_E}\tan L\sec L} \over {{R_N} + h}}} & 0 & { - \displaystyle{{{v_E}\sec L} \over {{{({R_N} + h)}^2}}}} \cr 0 & 0 & 0 \cr } } \right],{{ F}_{{G_{12}}}} = \left[ {\matrix{ 0 & {\displaystyle{1 \over {{R_M} + h}}} & 0 \cr {\displaystyle{{\sec L} \over {{R_N} + h}}} & 0 & 0 \cr 0 & 0 & 0 \cr } } \right]$

${{{F}}_{{G_{21}}}} = \left[ {\matrix{ {\left (2{\omega _{{\rm ie}}}({v_U}\sin L + {v_N}\cos L ) + \displaystyle{{{v_E}{v_N}} \over {{R_N} + h}}*{{\sec }^2}L\right)} & 0 & {\displaystyle{{{v_E}{v_U} - {v_E}{v_N}\tan L} \over {{{({R_N} + h)}^2}}}} \cr { - {v_E}\left(2{\omega _{{\rm ie}}}\cos L + \displaystyle{{{v_E}} \over {{R_N} + h}}{{\sec }^2}L\right)} & 0 & {\left(\displaystyle{{{v_N}{v_U}} \over {{{({R_M} + h)}^2}}} + \displaystyle{{v_E^2\tan L} \over {{{({R_N} + h)}^2}}}\right)} \cr { - 2{\omega _{{\rm ie}}}\sin L{v_E}} & 0 & { - \left(\displaystyle{{v_N^2} \over {{{({R_M} + h)}^2}}} + \displaystyle{{v_E^2} \over {{{({R_N} + h)}^2}}}\right)} \cr } } \right]$

${{ F}_{{G_{22}}}} = \left[ {\matrix{ {\displaystyle{{{v_N}\tan L - {v_U}} \over {{R_N} + h}}} & {\left(2{\omega _{{\rm ie}}}\sin L + \displaystyle{{{v_E}} \over {{R_N} + h}}\tan L\right)} & {\left(2{\omega _{{\rm ie}}}\cos L + \displaystyle{{{v_E}} \over {{R_N} + h}}\right)} \cr { - 2\left({\omega _{{\rm ie}}}\sin L + \displaystyle{{{v_E}} \over {{R_N} + h}}\tan L\right)} & { - \displaystyle{{{v_U}} \over {{R_M} + h}}} & { - \displaystyle{{{v_N}} \over {{R_M} + h}}} \cr {2\left({\omega _{{\rm ie}}}\cos L + \displaystyle{{{v_E}} \over {{R_N} + h}}\tan L\right)} & {\displaystyle{{2{v_N}} \over {{R_M} + h}}} & 0 \cr } } \right]$

${{ F}_{{G_{31}}}} = \left[ {\matrix{ 0 & 0 & {\displaystyle{{{v_N}} \over {{{({R_M} + h)}^2}}}} \cr { - {\omega _{{\rm ie}}}\sin L} & 0 & { - \displaystyle{{{v_E}} \over {{{({R_N} + h)}^2}}}} \cr {\left({\omega _{{\rm ie}}}\cos L + \displaystyle{{{v_E}\,{{\sec }^2}L} \over {{R_N} + h}}\right)} & 0 & { - \displaystyle{{{v_E}\tan L} \over {{{({R_N} + h)}^2}}}} \cr } } \right],{{F}_{{G_{32}}}} = \left[ {\matrix{ 0 & { - \displaystyle{1 \over {{R_M} + h}}} & 0 \cr { - \displaystyle{1 \over {{R_N} + h}}} & 0 & 0 \cr { - \displaystyle{{\tan L} \over {{R_N} + h}}} & 0 & 0 \cr } } \right]$

${{{F}}_{{G_{33}}}} = \left[ {\matrix{ 0 & {{\omega _{{\rm ie}}}\sin L + \displaystyle{{{v_E}} \over {{R_N} + h}}\tan L} & { - {\omega _{{\rm ie}}}\cos L - \displaystyle{{{v_E}} \over {{R_N} + h}}} \cr { - {\omega _{{\rm ie}}}\sin L - \displaystyle{{{v_E}} \over {{R_N} + h}}\tan L} & 0 & { - \displaystyle{{{v_N}} \over {{R_M} + h}}} \cr {{\omega _{{\rm ie}}}\cos L + \displaystyle{{{v_E}} \over {{R_N} + h}}} & {\displaystyle{{{v_N}} \over {{R_M} + h}}} & 0 \cr } } \right].$

${{ G}_G}(t)$ is the system noise driver array, the parameters of each variable in the mat-rix are shown in Eq. (10);

(10) $${{ G}_G}(t) = \left[ {\matrix{ {{0_{3 \times 3}}} & {{0_{3 \times 3}}} \cr {{0_{3 \times 3}}} & {C_b^n} \cr {C_b^n} & {{0_{3 \times 3}}} \cr {{0_{6 \times 3}}} & {{0_{6 \times 3}}} \cr } } \right]. $$

${{{W}}_G}(t) = {[{w_{gx}},{w_{gy}},{w_{gz}},{w_{ax}},{w_{ay}},{w_{az}}]^{\rm T}}$ is the system noise, where ${w_{gx}},{w_{gy}},{w_{gz}}$ is the white noise of the gyroscope along the $x,y,z$ axes of the rocket; ${w_{ax}},{w_{ay}},{w_{az}}$ is the white noise of the accelerometer along the $x,y,z$ axes of the rocket.

The velocity information solved by MEMS-SINS is denoted as $[{v_{{E_{SINS}}}};{v_{{N_{SINS}}}};{v_{{U_{SINS}}}}]$ and the position information as $[{L_{SINS}};{\lambda _{SINS}};{h_{SINS}}]$, and the velocity information solved by GNSS is denoted as $[{v_{{E_{GNSS}}}};{v_{{N_{GNSS}}}};{v_{{U_{GNSS}}}}]$ and the position information is denoted as $[{L_{GNSS}};{\lambda _{GNSS}};{h_{GNSS}}]$. By taking the difference between these two as the measurement information, the filtered measurement equations are established as shown in Eq. (11) (Hu et al., 2020a; Yang & Zhao, 2024).

(11) $${ Z} = { HX} + {{{ V}}_k} = \left[ {\matrix{ {{{{ H}}_{p,k}}} \cr {{{{ H}}_{v,k}}} \cr } } \right]{ X} + \left[ {\matrix{ {{{{ V}}_{p,k}}} \cr {{{{ V}}_{v,k}}} \cr } } \right].$$

${Z}$ is the value of the measurement information;

${{{H}}_{p,k}} = [diag({R_M},{R_N}\cos L,1),{0_{3 \times 12}}]$ and ${{{H}}_{v,k}} = [{0_{3 \times 3}},{I_{3 \times 3}},{0_{3 \times 9}}]$ is the measurement transfer matrix; ${{{V}}_{p,k}}$ and ${{{V}}_{v,k}}$ is the measurement noise of GNSS.

According to the continuous state Eq. (8) and the measurement Eq. (11), the set of filtering equations after discretization is shown in Eqs. (12) to (16) (Zhao et al., 2022).

The one-step prediction state equation is as follows:

(12) $${{{\hat { X}}}_{k/k - 1}} = {{{\phi }}_{k,k - 1}}{{{\hat { X}}}_{k - 1}}.$$

The estimated state equation is as follows:

(13) $${{{\hat { X}}}_k} = {{{\hat { X}}}_{k/k - 1}} + {{{K}}_k}({{{Z}}_k} - {{{H}}_k}{{{\hat { X}}}_{k/k - 1}}).$$

The filter gain calculation equation is as follows:

(14) $${{{K}}_k} = {{{P}}_{k/k - 1}}{{H}}_k^{\rm T}{({{{H}}_K}{{{P}}_{k/k - 1}}{{H}}_k^{\rm T} + {{{R}}_k})^{ - 1}}.$$

The one-step prediction mean-variance equation is as follows:

(15) $${{{P}}_{k/k - 1}} = {{{\phi }}_{k,k - 1}}{{{P}}_{k - 1}}{{\phi }}_{k,k - 1}^{\rm T} + {{{\bf \Gamma }}_{k - 1}}{{ Q}_{k - 1}}{{\bf \Gamma} }_{k - 1}^{\rm T}.$$

The filtering estimation mean square error equation is as follows:

(16) $${{{P}}_k} = ({ I} - {{{K}}_k}{{{H}}_k}){{{P}}_{k/k - 1}}{({ I} - {{{K}}_k}{{{H}}_k})^{\rm T}} + {{{K}}_k}{{{R}}_k}{ K}_k^{\rm T}.$$

In the above filtering equation, ${{{\phi }}_{k,k - 1}} = diag(1,1,1,1,1,1,1,1,1,1,1,1,1,1,1) - {{{F}}_G}\Delta t$ is the one-step transfer matrix, ${{{K}}_k}$ is the filtering gain, ${{{Q}}_k}$ is the variance matrix of the MEMS inertial device noise ${{{W}}_G}$, and ${{{R}}_k}$ is the variance matrix of the GNSS measurement noise ${{{V}}_k}$.

According to Eqs. (12) to (16), the navigation error estimation of inertial navigation can be realized by setting the initial state of the filter as well as the filtering parameters, and combining the state output at the moment k as well as the measurement output, and the navigation error correction can be carried out at the next moment to improve the accuracy of the integrated navigation system. However, in the case of MEMS_SINS, the deviations in structural design and manufacturing processes complicate the modeling of the inertial navigation system. Consequently, accurately designing the filtering parameters becomes challenging, which adversely affects the accuracy and robustness of the integrated navigation system based on EKF technology. In particular, the short-term filter convergence performance receives a large impact (Fernandes et al., 2023; Yang et al., 2021).

### Adaptive parameter reconstruction method

Currently, the navigation system of the guided rocket requires self-alignment of the initial navigation parameters prior to launch (Zhu et al., 2022). This article considers the significant performance disparities among various MEMS inertial devices and considers the application environment of MEMS-SINS/GNSS on the rocket. We propose an extended Kalman filter method with adaptive reconstruction parameters. During the self-alignment phase of the rocket’s navigation system, we perform adaptive real-time estimation of the filter parameters and reset these parameters before launch. This approach aims to achieve precise design of the navigation filter parameters, thereby enhancing the accuracy and dynamic performance of the rocket navigation system while ensuring compliance with real-time requirements.

The filter parameter ${{{Q}}_k}$, which characterizes the noise of inertial devices, plays a crucial role in determining the convergence speed and solution accuracy of the EKF. It is one of the most significant parameters in filter design (Luo, Guo & Chen, 2023; Zhao et al., 2022). The varying noise characteristics of different inertial devices are a key factor contributing to suboptimal EKF filtering performance. In designing the filter parameter ${{{Q}}_k}$, it is assumed that there is no correlation among the three sensors, as expressed in Eq. (17).

(17) $${{{Q}}_k} = diag(N_{AR{W_{\omega x}}}^2,N_{AR{W_{\omega y}}}^2,N_{AR{W_{\omega z}}}^2,N_{AR{W_{Ax}}}^2,N_{AR{W_{Ay}}}^2,N_{AR{W_{Az}}}^2).$$

${N_{ARW}}$ is the random wandering coefficient of the inertial device.

According to Eq. (14), the accurate design of the EKF filter parameter matrix ${{{Q}}_k}$ can be achieved upon the completion of the real-time estimation of the random walk coefficient. During the self-alignment stage of the rocket navigation system, this real-time estimation is accomplished by modeling and analyzing the output data from the inertial device, thereby facilitating the adaptive reconstruction of the EKF navigation filter parameters. The implementation process of the proposed method is outlined as follows.

(1) The real-time output of the inertial device during the self-alignment process of the rocket navigation system is recorded to create a sample sequence for estimating the random walk coefficient. This sample sequence is presented in Eq. (15).
(18) $$\left[ {{{\bar \Omega }_1}({\tau _i}),{{\bar \Omega }_2}({\tau _i}),{{\bar \Omega }_3}({\tau _i}), \cdot \cdot \cdot ,{{\bar \Omega }_{{N_i}}}({\tau _i})} \right]$$
${\tau _i}$ is the data update period.
${N_i}$ is the number of MEMS-SINS output data points.
(2) Calculate the variance of the data at the sampling time ${\tau _i}$, as shown in Eq. (19):
(19) $${\hat \sigma _A}({\tau _i}) = \sqrt {\displaystyle{1 \over {2*({N_i} - 1)}}*\sum\limits_{k = 1}^{N - 1} {{{({{\bar \Omega }_{k + 1}}({\tau _i}) - {{\bar \Omega }_k}({\tau _i}))}^2}} }.$$
(3) Update data update period ${\tau _{i + 1}} = 2*{\tau _i}$, Update data Length ${N_{i + 1}} = {N_i}/2$, repeat steps 1 and 2
(4) By continuously varying the sampling period of the output data from the MEMS inertial device, a correlation array is generated as shown in Eq. (20), which relates the sampling period to the data variance.
(20) $${\hat \sigma _{{A_i}}} = f({\tau _i}).$$
(5) In the analysis of noise specific to MEMS inertial devices, the logarithm of the variance of angle/velocity random wander performance *vs*. sampling time ideally forms a straight line with a slope of −0.5. Therefore the correlation array according to Eq. (20) is fitted to find the curve that is closest to the slope of −0.5, as shown in Eq. (21).
(21) $$\eqalign{ & count = find(\min (\log (\Delta {\sigma _i})/\log (\Delta {\tau _i}) + 0.5)) \cr  & \log b = \log (\Delta {\sigma _{count}}) + 0.5*\log (\Delta {\tau _{count}}) \cr}$$
  where $\log b$ is a constant term in the linear fit curve.
(6) The computationally estimated inertial device random wandering is shown in Eq. (22).
(22) $${N_{ARW}} = {10^{\log b}}$$

### Summary of the proposed algorithm

**Figure 1:**
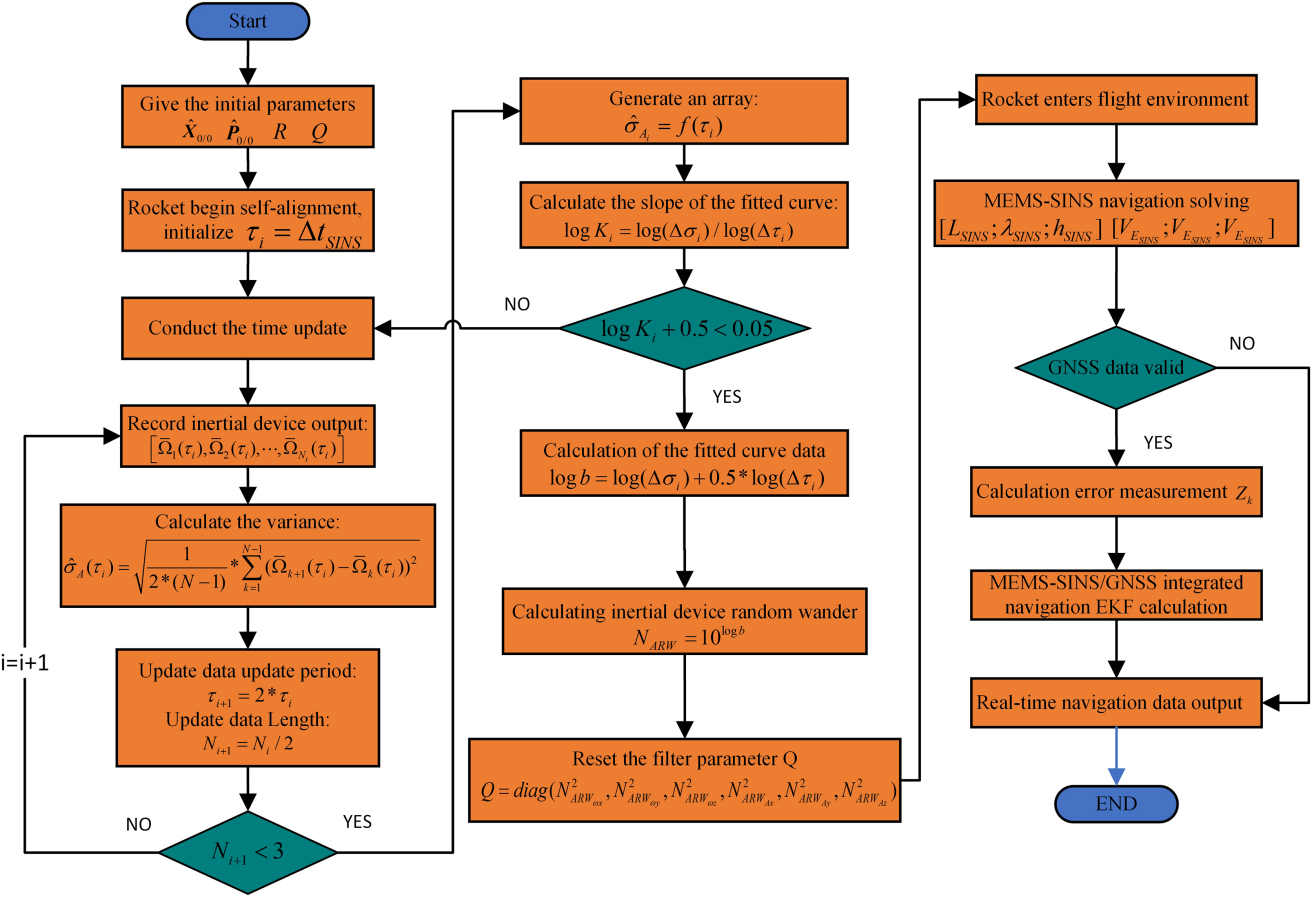
The flowchart of the adaptive reconfigurable extended Kalman filter.

The flow chart of the AREKF algorithm is illustrated in Fig. 1, In the figure, the MEMS-SINS navigation calculation process is shown in Eqs. (1), (2) and (3); The calculation of the error measurement value is presented in Eq. (11); The EKF filtering method for SINS/GNSS integrated navigation is outlined in Eqs. (12) through (16). In our proposed AREKF, for the rocket application environment, the Extended Kalman Filter(EKF) under the high-order state parameters is adopted, and the accurate calculation of filter parameters is carried out based on the rocket navigation alignment process, which realizes the adaptive reconstruction of filter parameters in the rocket flight, and this method not only retains the advantages of the EKF algorithm with small computational volume, but also greatly improves the convergence speed and navigation accuracy of the integrated navigation and has better navigation performance.

## Digital simulation

In this section, we evaluate the performance of the proposed AREKF by simulating the 6D trajectory of a rocket through numerical integration of the MEMS-SINS/GNSS navigation system. To quantitatively assess the effectiveness and superiority of the proposed AREKF algorithm, we compare it against several representative methods, including the conventional EKF, the invariant extended Kalman filter (IEKF) (Tang et al., 2024), the maximal soria criterion extended Kalman filter (MVC-EKF) (Zhao et al., 2022), and the matrix Lie group of double direct isometries extended Kalman filter (SE2(3)-EKF) (Luo, Guo & Chen, 2023).

### MEMS-SINS/GNSS noise model

In the inertial navigation equation of state model presented in Eq. (5), ${{{W}}_G}\sim(0,{{{Q}}_k})$ represents the process noise of the MEMS-SINS/GNSS integrated navigation system. It is assumed that the process noise is white and independent. Here, ${{{Q}}_k}$ is the noise covariance matrix in the EKF equation, which can vary significantly due to differences in inertial navigation devices and environmental conditions. In the context of GNSS navigation, as stated in Eq. (8), ${{{V}}_k}\sim(0,{{ R}_k})$ signifies the measurement noise of the MEMS-SINS/GNSS integrated navigation system, with ${{{R}}_k}$ serving as the measurement noise covariance matrix, also assuming white and independent process noise. Furthermore, for the GNSS navigation output model in Eq. (8). The objective of our proposed method is to evaluate navigation performance under varying process noise conditions. Consequently, the noise model is uniform across all other noise models. The statistical model of the noise used in the simulation is detailed in Table S1, and the characteristics of the process noise are defined as Gaussian distributions with significantly increased covariance to simulate real-world noise variations.

### Simulation of rocket trajectory

**Figure 2:**
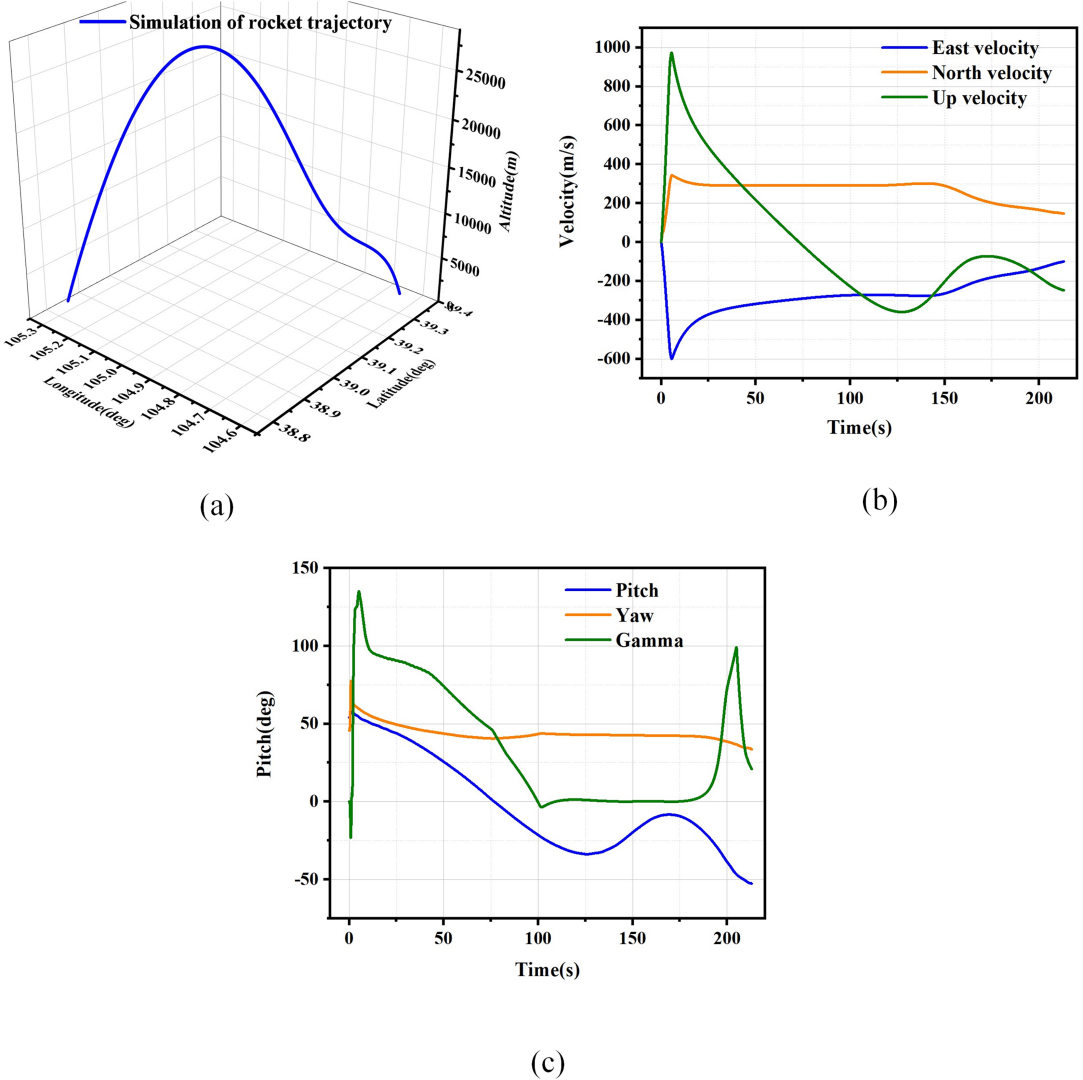
Rocket location information. (A) The true trajectory of rocket. (B) The true velocity of the rocket. (C) The true altitude of rocket.

The high overload and real-time requirements of the rocket flight process present challenges for the design of MEMS-SINS/GNSS combined navigation systems. The high overload and real-time requirements of the rocket flight process present challenges for the design of MEMS-SINS/GNSS combined navigation systems. In this context, the term ‘high overload conditions’ refers to the significant overload and extensive maneuvering flight environment encountered by the rocket. Figure 2 present the theoretical flight trajectory information utilized in the numerical simulation, encompassing position, velocity, and attitude data. Notably, within the theoretical trajectory, the velocity change reaches 900 m per second within a span of 20 s, thereby satisfying the criteria for high overload conditions. Additionally, the changes in pitch and roll angles exceed 90 degrees, aligning with the requirements for large maneuvering flight conditions. Conducting numerical simulations based on the trajectory conditions illustrated in the figures effectively validates the performance of the integrated navigation filtering algorithm under high overload conditions.

### Simulation result comparison with different algorithms

We conducted a comprehensive simulation of the MEMS-SINS/GNSS integrated navigation system to validate the performance of the proposed AREKF under conditions of unknown process noise. The settings for the MEMS-SINS/GNSS noise model are detailed in Table S1, and the real trajectories in the simulation are shown in Fig. 2. The update rates for the SINS and GNSS are set at 400 and 10 Hz, respectively, the initial parameter configuration of the filter is shown in Table S2.

As demonstrated in Table S2, ${L_0},{\lambda _0},{h_0}$ and ${v_{{E_0}}},{v_{{N_0}}},{v_{{U_0}}}$ represents the initial navigation position information and velocity, which can be provided by ground equipment prior to the rocket launch. ${\vartheta _0},{\psi _0},{\gamma _0}$ denotes the initial attitude information, obtained through initial alignment before launch. Both L and pitch serve as the initial values for inertial integration prior to navigation launch. ${X_{0/0}}$ and ${P_{0/0}}$ denote the mean and variance of the filter state parameters, respectively. These parameters are determined based on the measurement accuracy of the ground state system and the stability of the inertial navigation alignment process. ${Q_k}$ and ${R_k}$ represent the state noise parameter and the satellite navigation measurement noise, respectively, and are determined according to the noise characteristics of the inertial devices and satellite navigation positioning.

To compare the performance differences between the proposed AREKF and existing methods,

We define the performance metrics for stability, convergence speed, and estimation accuracy of the combined navigation filtering. These metrics are defined as follows:

(a) The combined navigation filter stabilization coefficient $\lambda$ is defined as the square of the ratio of the absolute value of the navigation solution error squared to the variance of the error covariance matrix, as illustrated in Eq. (23).
(23) $$\lambda = \displaystyle{{{{(x_k^s - \hat x_k^s)}^2}} \over {{P_{{x_k}}}}}*100.$$
(b) Set the convergence time $\tau$ for the combined navigation filter, define the error threshold $\varepsilon$, and record the timestamp when the filtering error first falls below the specified threshold.
(c) we selected the Root Mean Square Error (RMSE) and Average Root Mean Square Error (ARMSE) of the attitude, velocity, and position over time for the integrated MEMS-SINS/GNSS navigation system. These metrics are defined as shown in Eqs. (24) and (25) (Feng et al., 2021).

(24) $$RMSE = \sqrt {\displaystyle{1 \over M}\sum\limits_{s = 1}^M {{{(x_k^s - \hat x_k^s)}^2}} }$$

(25) $$ARMSE = \sqrt {\displaystyle{1 \over {MT}}\sum\limits_{k = 1}^T {\sum\limits_{s = 1}^M {{{(x_k^s - \hat x_k^s)}^2}} } }$$

where $x_k^s$ and $\hat x_k^s$ denote the true and the estimated value of attitude, velocity and position at time $k$ of the $sth$ Monte Carlo run, respectively. $M$ denotes the number of Monte Carlo run. $T$ represents the total simulation samples during simulation time (Feng et al., 2021).

**Figure 3:**
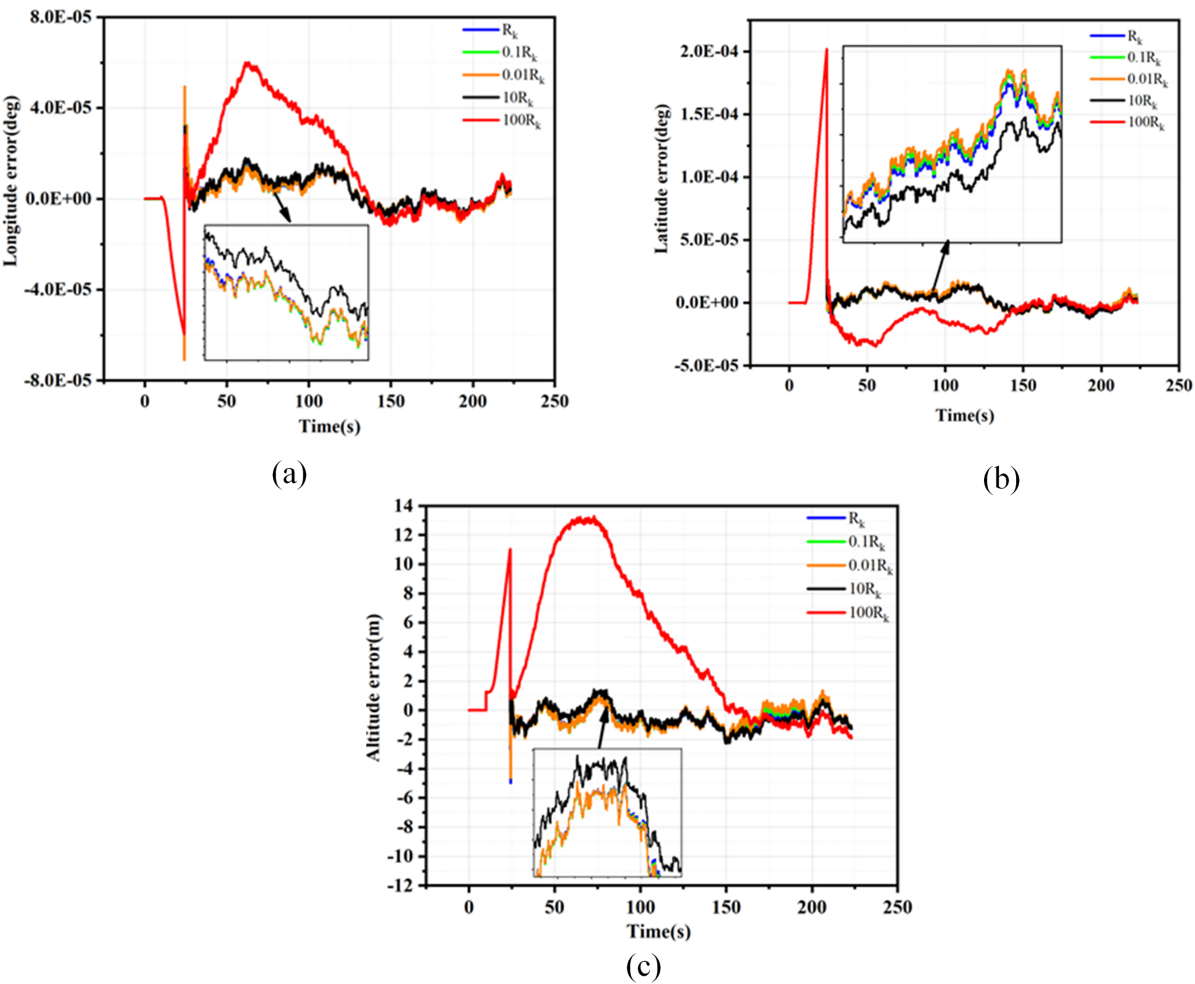
Position error for different Rk. (A) Latitude errors for different Rk; (B) Longitude errors for different Rk; (C) Altitude errors for different Rk.

**Figure 4:**
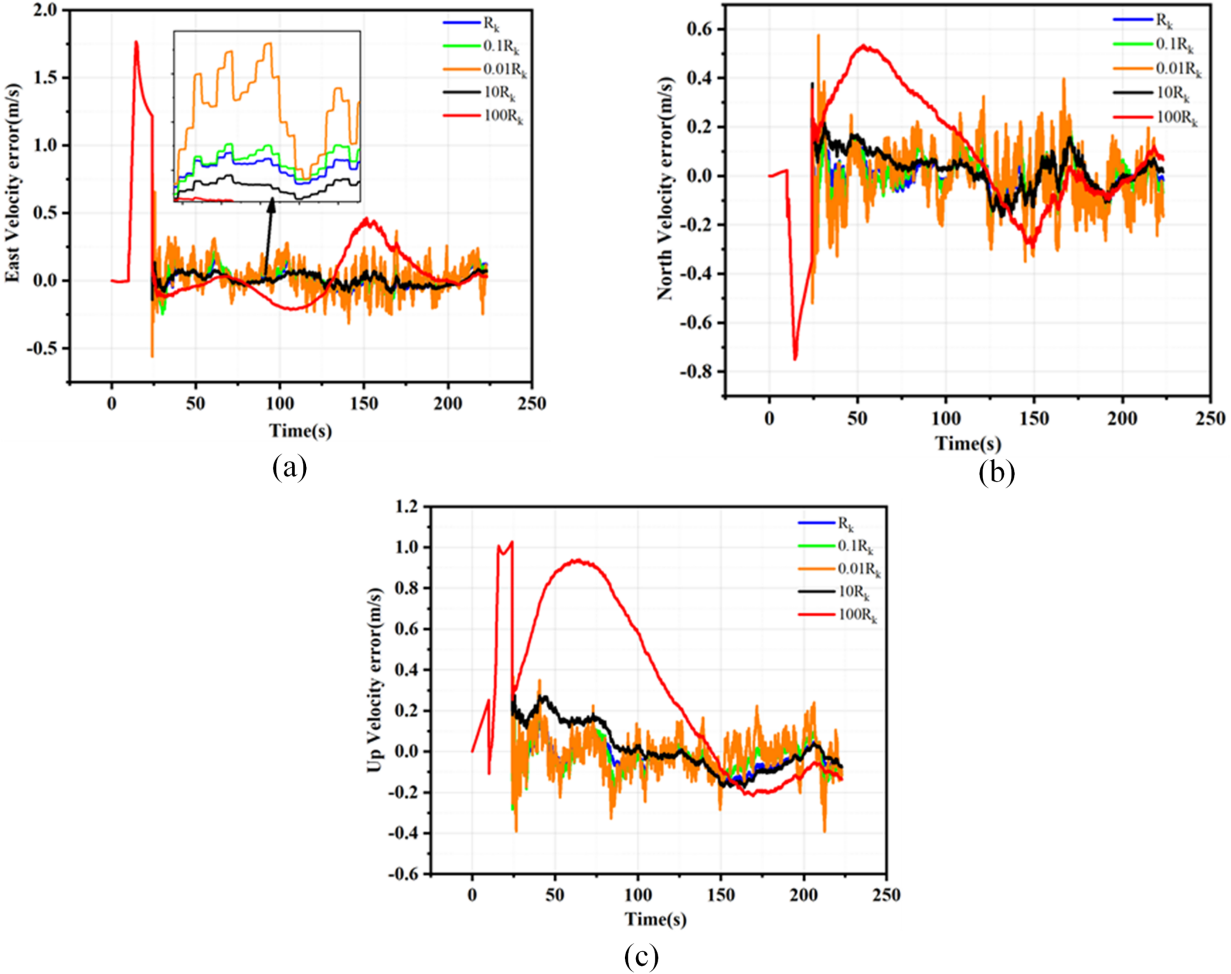
Velocity error for different Rk. (A) East velocity errors for different Rk; (B) North velocity errors for different Rk; (C) Up velocity errors for different Rk.

**Figure 5:**
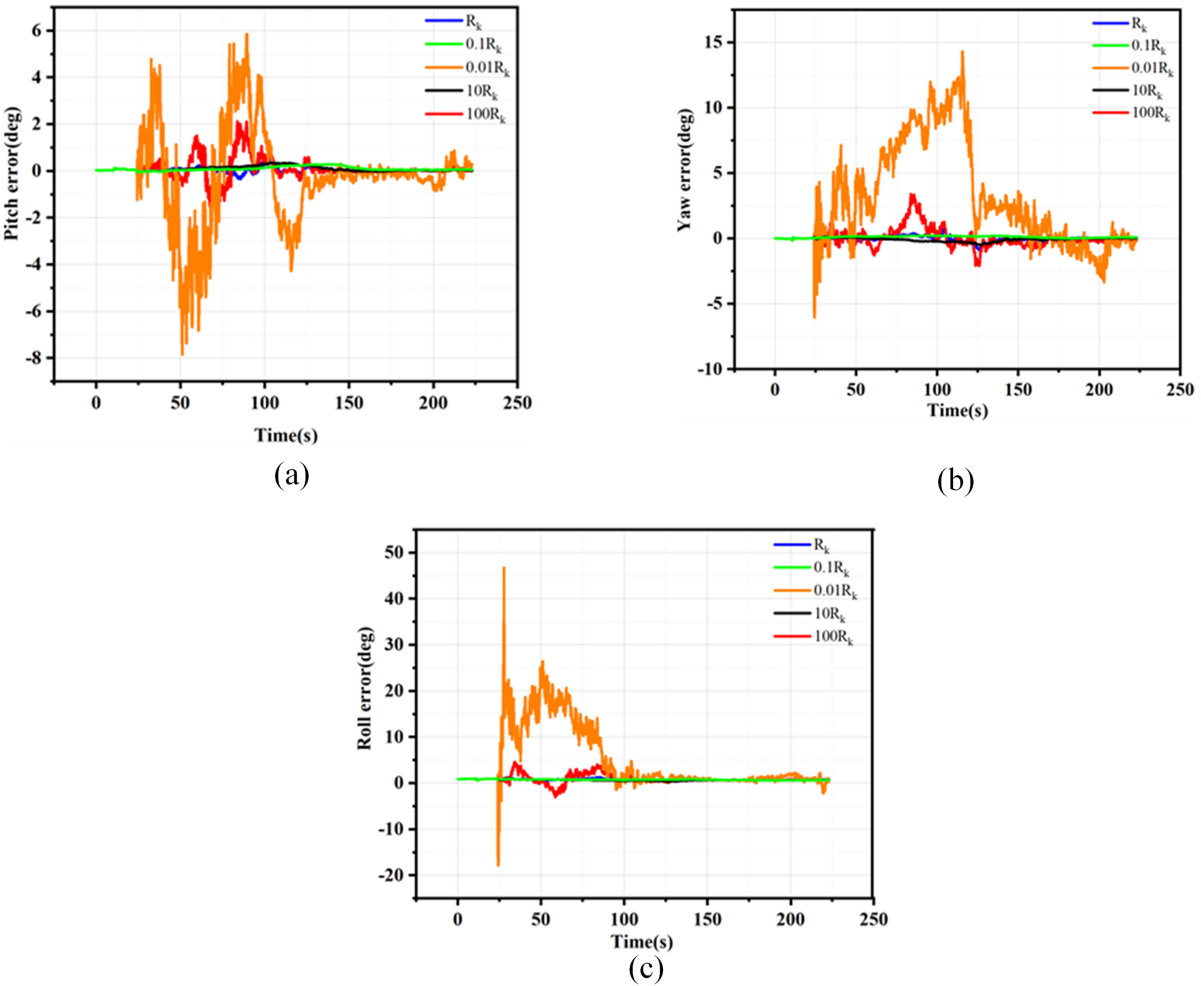
Attitude error for different Rk. (A) Pitch errors for different Rk. (B) Yaw errors for different Rk. (C) Roll errors for different Rk.

As a key filtering parameter in the EKF method, the design accuracy of the matrix ${R_k}$ significantly influences the filtering performance of combinatorial navigation. In this article, we conduct 150 Monte Carlo simulations based on the traditional EKF scheme, utilizing various filtering parameters ${R_k}$ set according to the simulation parameters outlined in Tables S1 and S2. This approach aims to verify the impact of R on filtering performance. The simulation settings for the filtering parameter ${R_k}$ are detailed in Table S3, while the velocity, position, and attitude root mean square error (RMSE) curves resulting from the Monte Carlo simulations are presented in Figs. 3 to 5.

As evidenced by the results presented in Figs. 3 to 5, when the filtering parameter ${R_k}$ is maintained within the same order of magnitude as the measurement noise characteristics of GNSS, the RMSE deviation of the navigation information remains insignificant. Consequently, it is crucial to keep the filtering parameter ${R_k}$ within one order of magnitude of the GNSS measurement noise characteristics during the filtering process. This article utilizes the EKF method under loosely coupled conditions and employs the position dilution of precision (PDOP) to preliminarily assess the validity of GNSS measurement data. When the PDOP value is within the specified threshold range, integrated navigation filtering of the GNSS output data is conducted. The noise fluctuation range while filtering GNSS navigation data remains within one order of magnitude of the threshold limit, thus determining the appropriate filtering parameter ${R_k}$.

**Figure 6:**
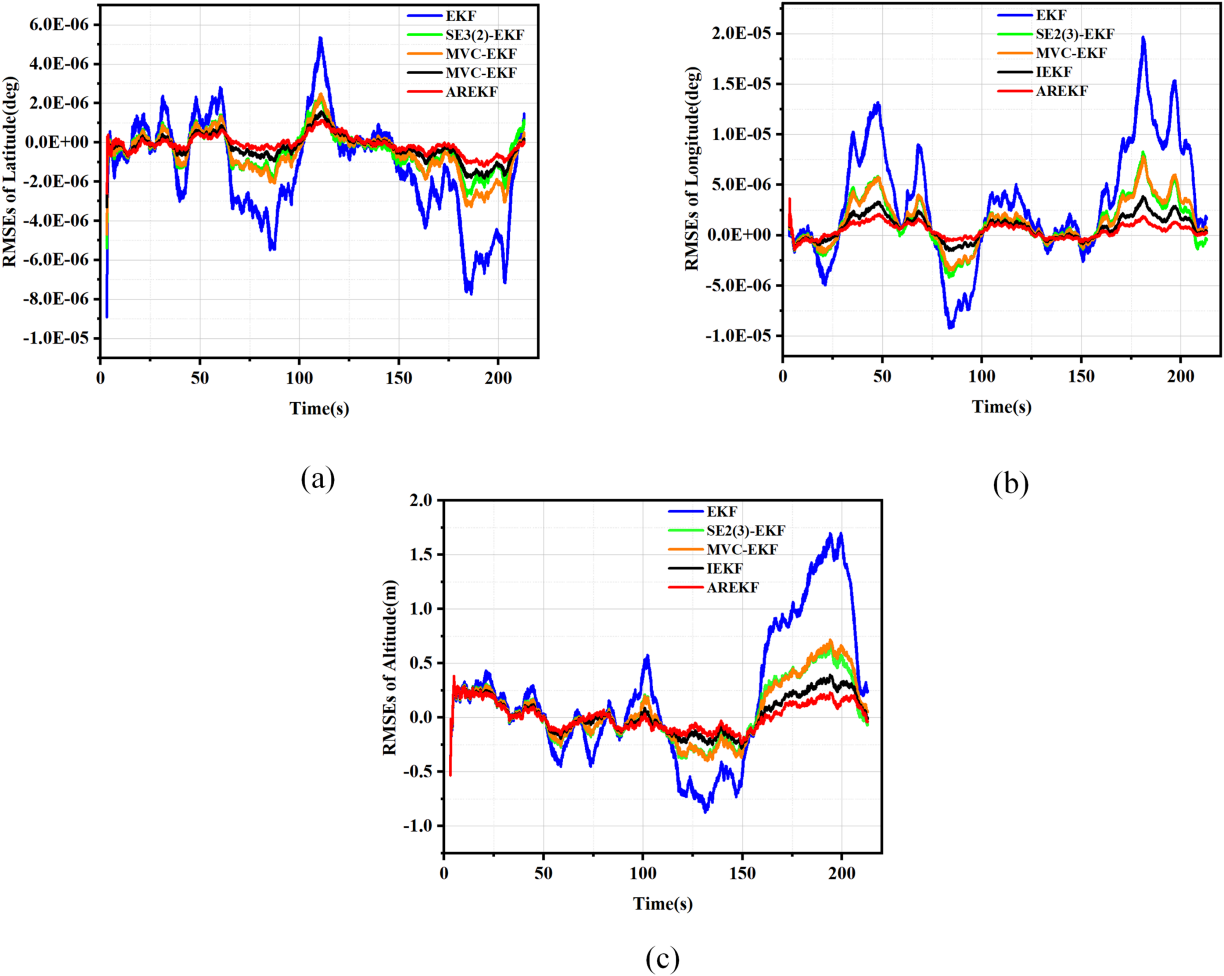
RMSE plots for position. (A) RMSEs in latitude; (B) RMSEs in longitude; (C) RMSEs in altitude.

**Figure 7:**
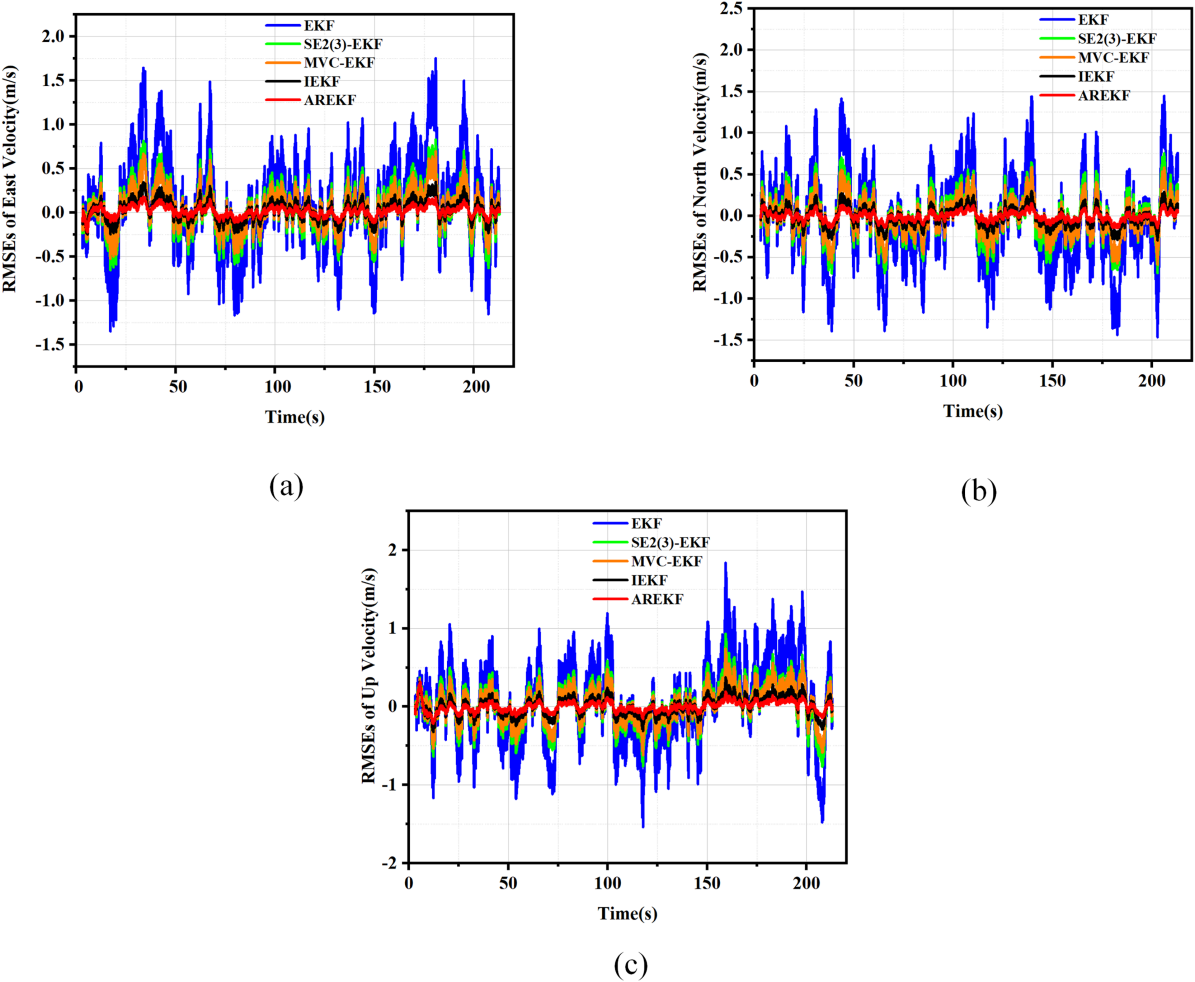
RMSE plots for velocity. (A) RMSEs in east velocity; (B) RMSEs in north velocity; (C) RMSEs in up velocity.

**Figure 8:**
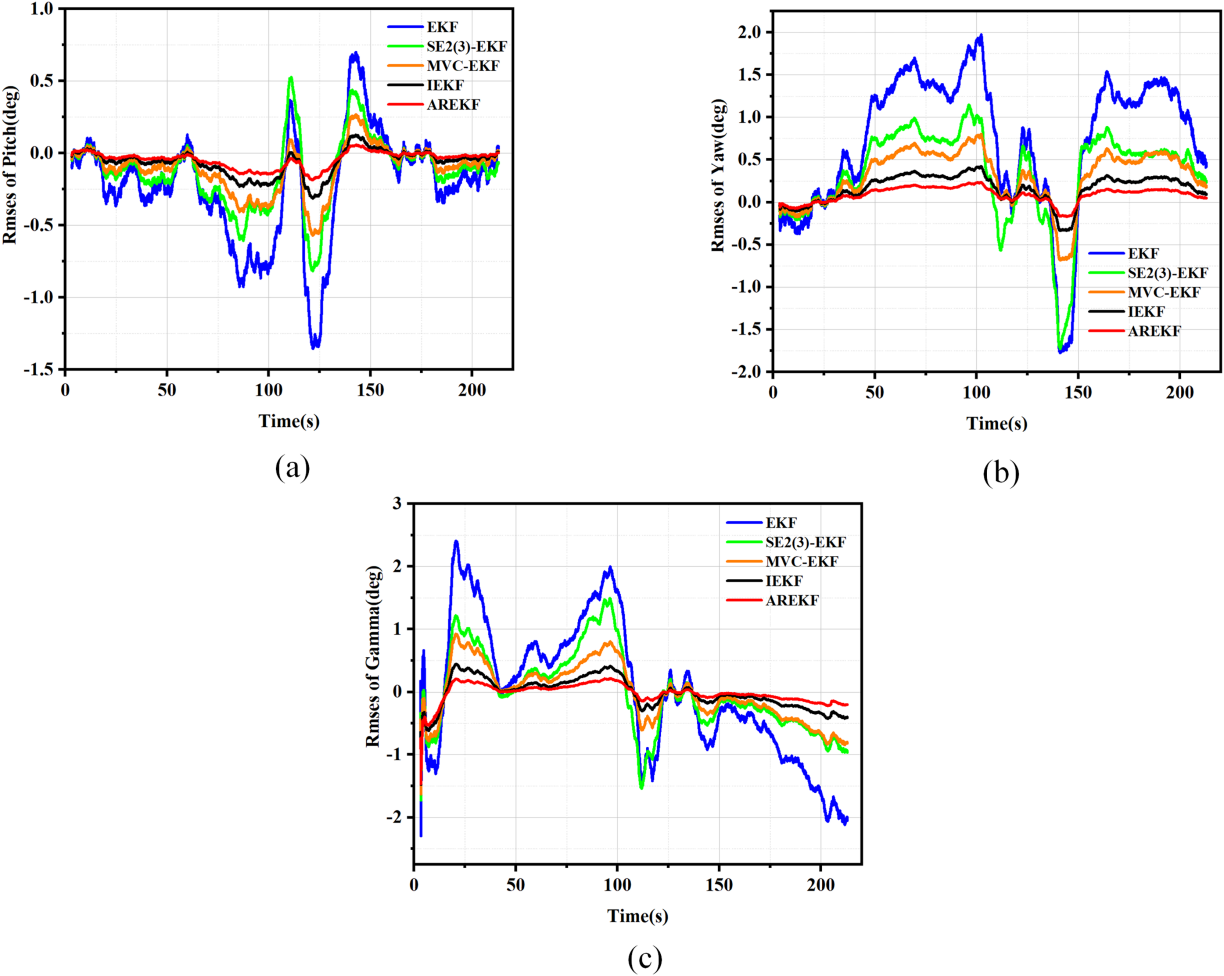
RMSE plots for attitude. (A) RMSEs in pitch; (B) RMSEs in yaw; (C) RMSEs in roll.

By comparing the proposed AREKF with the conventional EKF, IEKF (Ibrahim et al., 2023), MVC-EKF (Zhao et al., 2022), and SE2(3)-EKF (Luo, Guo & Chen, 2023), we validate the superior performance of the AREKF. In the design of the loosely coupled MEMS-SINS/GNSS integrated navigation filtering algorithm, the performance of five filtering algorithms is evaluated through 150 Monte Carlo simulations. The RMSE plots for velocity, position, and attitude of the five filtering methods are presented in Figs. 6 to 8.

According to the simulation results presented in the figure, the performance metrics for position, velocity, and attitude were compared under various algorithm simulation conditions. The summary of the comparison of the test results is as follows:

(a) In terms of filtering stability, considering that the variance thresholds of position, velocity, and attitude are relatively fixed, the stability size depends on the error value of navigation solving. According to the results presented in the figure, it is evident that the AREKF method exhibits superior performance in the presence of inertial equipment noise characteristic deviations. Notably, the attitude filtering stability achieved by the AREKF method is significantly higher than that of existing filtering methods.
(b) In terms of filtering convergence time, the performance of several filtering methods exhibits minimal variation. This observation primarily arises from the fact that when navigation data is updated more frequently, the differences in convergence speed among these methods become less pronounced.
(c) In terms of filtering estimation accuracy, the AREKF proposed in this article demonstrates a significant improvement over other filtering methods. Notably, both the velocity and attitude estimation accuracies are enhanced, with performance improvements exceeding twofold.

In summary, the AREKF method consistently exhibited superior characteristics compared to existing algorithms, primarily due to the mismatch between the default filtering parameters and the noise characteristics of inertial devices across different algorithms. The traditional EKF fails to adjust the default filtering parameters, resulting in the poorest performance under high-dynamic flight conditions. In contrast, the MVC-EKF and the SE2(3)-EKF address this inconsistency between filtering parameters and the noise characteristics of inertial devices through data preprocessing. The IEKF significantly enhances the robustness of the integrated navigation filtering system and mitigates the impact of variations in inertial device noise characteristics by alleviating the influence of state-dependent Jacobian matrices. The AREKF algorithm proposed in this article performs adaptive estimation of inertial device noise during navigation alignment in cases of abnormal default filtering parameter design, guiding and reconstructing the filtering parameters to achieve precise design of the filtering algorithm. This approach effectively addresses the significant changes in inertial device noise characteristics that occur under long-term storage conditions of rocket projectiles.

## Car-mounted experiments for SINS/GNSS integration navigation

In this section, the engineering validity and superiority of the algorithms in this article are verified through the experiments of the car-mounted MEMS-SINS/GNSS integrated navigation system.

### Different algorithmic computation time

Considering the engineering implementation of the algorithms, the Texas Instruments chip TMS320C6748 has been selected as the operational environment for all algorithms, and the computation time of the integrated navigation filtering algorithms has been recorded. The running times of various algorithms within a single step are presented in Table S4. Notably, the running time of the SE2(3)-EKF algorithm significantly exceeds the 2.5 ms sampling cycle time of the IMU, making it unsuitable for the real-time requirements of the rocket navigation system. Consequently, in the sports car test, only the navigation performance of the EKF, IEKF, MVC-EKF, and the proposed AREKF is evaluated.

### Experiment setup and description

**Figure 9:**
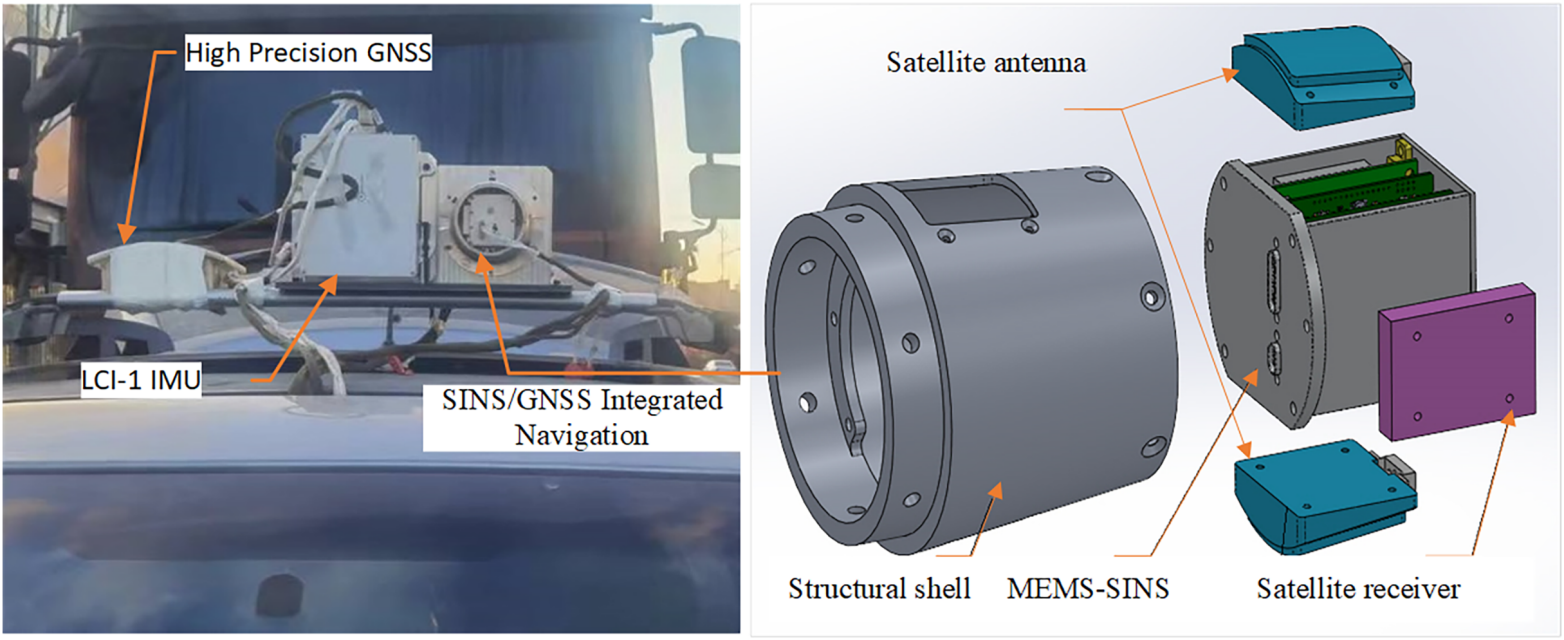
The car-mounted experimental platform.

The car-mounted experimental platform is depicted in Fig. 9, which includes a homemade MEMS-SINS/GNSS integrated navigation system alongside a high-precision integrated navigation system based on SINS/GNSS. The right section of the figure illustrates the structure of the homemade MEMS-SINS/GNSS integrated navigation system, while the high-precision integrated navigation system features an LCI-1 tactical-grade IMU and a high-precision GNSS device composed of the Propak satellite receiver and two antennas. This system is utilized to provide high-precision reference measurements for position, velocity, and attitude, achieving accuracies of 0.1 m, 0.05 m/s, and 0.01°, respectively. Table S5 outlines the sensor specifications of the homemade MEMS-SINS/GNSS integrated navigation system. The initial velocity and position for the filter are directly derived from the GNSS measurements, while the attitude information is obtained from the coarse alignment results of the SINS under heading binding. The initial parameter values for the filter are sourced from Table S2, with the process noise parameter settings adjusted to be twice as high as those specified in Table S3. This on-board system can be employed to test and verify the performance of the proposed filtering method in the presence of uncertain noise.

### Performance comparison with different algorithms

**Figure 10:**
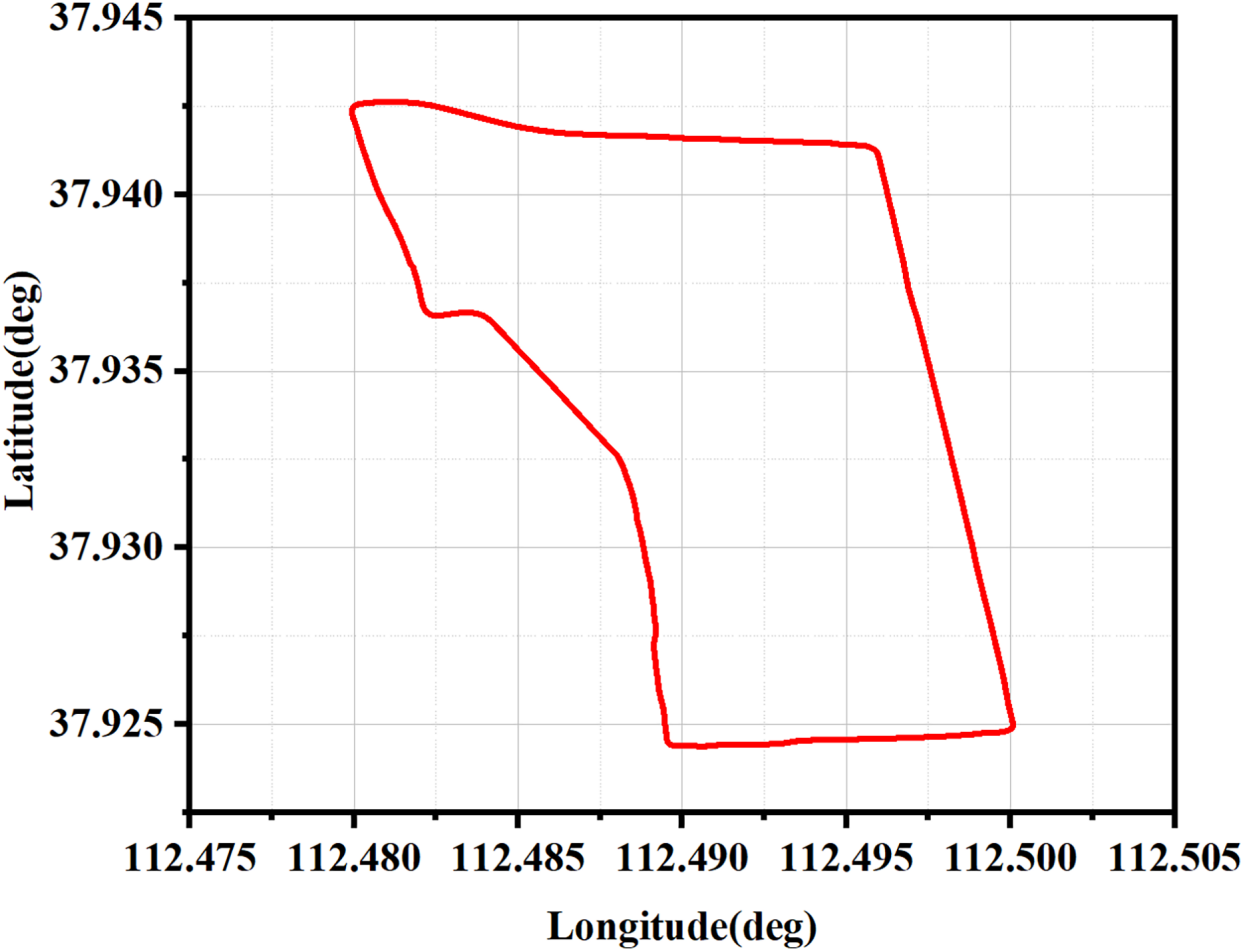
The true trajectory of the vehicle.

The trajectory of the car-mounted experiments is illustrated in Fig. 10, which serves to validate the performance of four distinct filtering algorithms.

**Figure 11:**
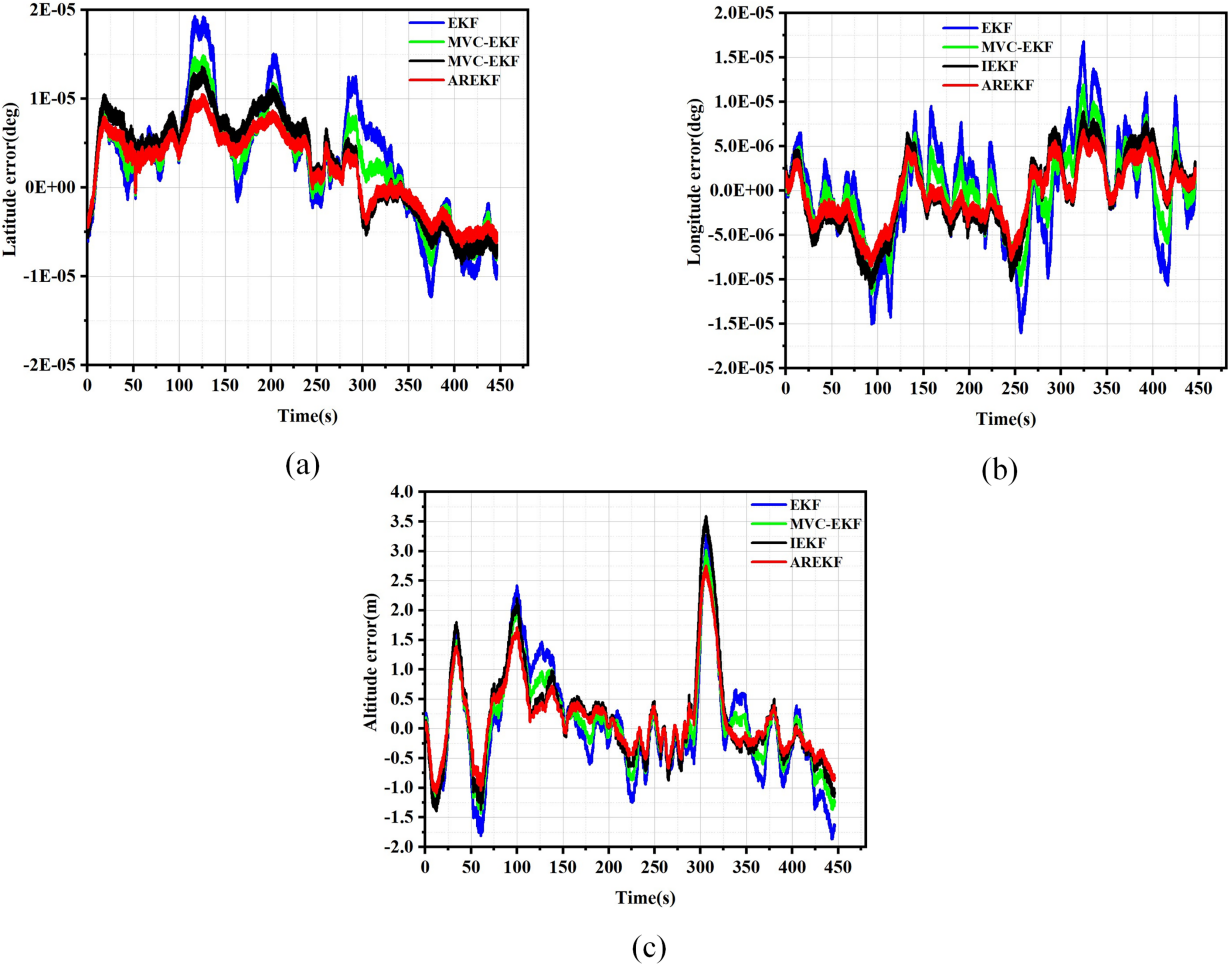
Position error. (A) Latitude error; (B) Longitude error; (C) Altitude error.

**Figure 12:**
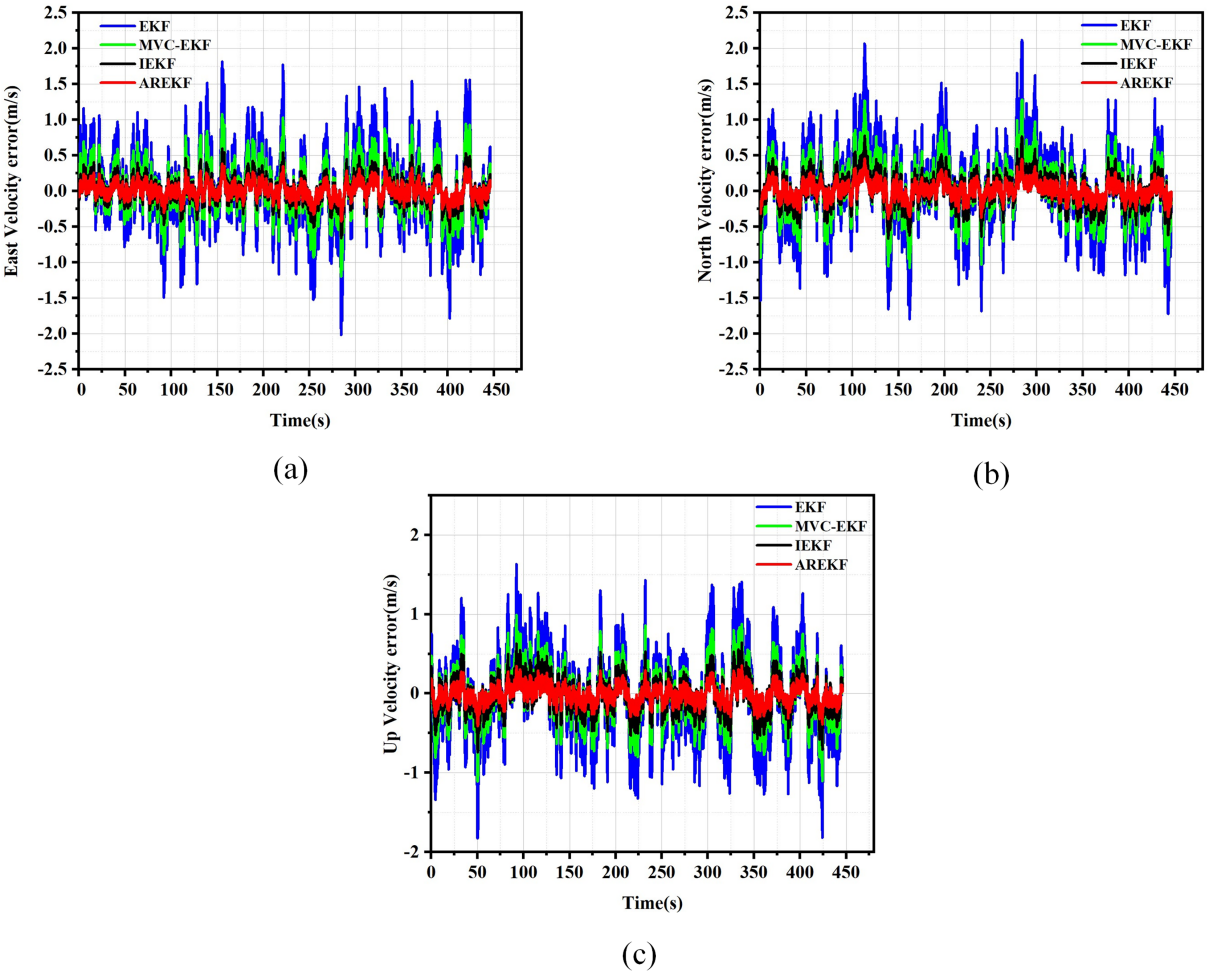
Velocity error. (A) East velocity error; (B) North velocity error; (C) Up velocity error.

**Figure 13:**
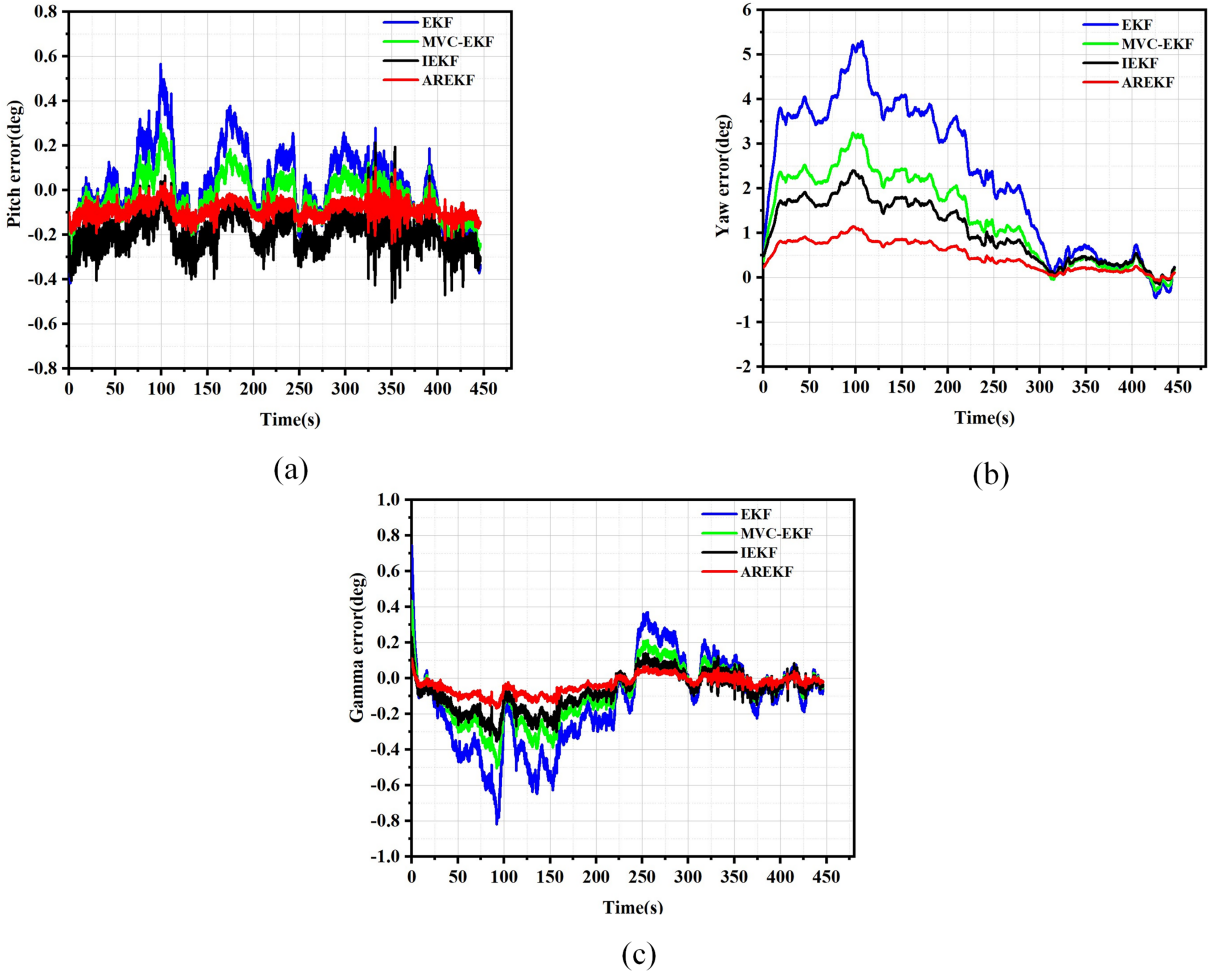
Attitude error. (A) Pitch error; (B) Yaw error; (C) Roll error.

The results of position, velocity, and attitude errors obtained from various algorithms after the car-mounted experiments are presented in Figs. 11 to 13.

To evaluate navigation performance in real-world conditions, the ground running car test serves as a means to validate the performance of the filtering algorithm. Based on the results obtained from the running car tests illustrated in Figs. 11 to 13, the following conclusions can be drawn:

(a) In terms of filtering stability, the low dynamic characteristics of the navigation system in the ground-based sports car validation result in a minimal difference between position filtering stability and velocity filtering stability. The variations among different filtering methods primarily manifest in attitude information. Notably, the AREKF method demonstrates a significant advantage in yaw angle filtering stability, providing superior filtering performance.
(b) The convergence times of the various algorithms are essentially equivalent regarding filter convergence time. This experimental result aligns with the conclusions drawn from the numerical simulations.
(c) In terms of filtering estimation accuracy, the position errors of various filtering methods are not significant. The AREKF method exhibits a slightly lower velocity error compared to the IEKF. However, the AREKF demonstrates superior performance regarding attitude error. The lateral maneuvering conditions experienced by the sports car contribute to the AREKF’s enhanced estimation accuracy, with attitude estimation accuracy improved by more than a factor of two.

To facilitate a detailed comparison of the filtering estimation accuracy across various filtering methods, Based on the definition of the performance metric ARMSE, the results of ten car tests were statistically analyzed for ARMSE, as presented in Table S6.

The statistical results in the table show that the proposed AREKF method yields lower ARMSE results in three areas: navigation position, velocity, and attitude, indicating higher computational accuracy and filtering stability. Compared to existing algorithms, the navigation performance has improved by over 30%.

The results of the car-mounted experiments indicate that Gaussian filtering is unsuitable for nonlinear systems with uncertain noise. Consequently, the EKF exhibits the poorest performance. In contrast, the MVC-EKF method demonstrates superior performance over the EKF, highlighting the effectiveness of data processing prior to fusion in mitigating uncertain noise. Furthermore, the IEKF enhances robustness to uncertain noise through the application of the weakened Jacobi matrix method. This article demonstrates that robustness to uncertain noise is significantly improved through parameter adaptive reconstruction derived from precise filter design, which in turn enhances navigation accuracy and dynamic performance. Therefore, considering the dynamic characteristics, accuracy, and computational efficiency of the rocket, we conclude that our proposed AREKF method effectively addresses the degradation of navigation performance due to noise uncertainty. It is capable of adapting to highly dynamic flight environments while achieving higher navigation accuracy without compromising the real-time performance of the rocket navigation system.

## Conclusion

In this article, a new adaptive parameter reconstruction extended Kalman filter is proposed, which exhibit strong robustness against the noise uncertainty under high overload of rockets. Initially, an extended Kalman filter is designed based on the higher-order state of the rocket flight environment. Subsequently, real-time estimation of IMU noise parameters is conducted during the rocket navigation alignment phase, enabling the adaptive reconstruction of filter parameters. This approach enhances navigation accuracy while accommodating the high overload and real-time performance requirements of rockets. Digital simulations and on-board test results indicate that the AREKF outperforms existing EKF filtering algorithms by over 30% in the presence of uncertain noise, thereby confirming its superior navigation accuracy in the high overload and real-time contexts of rocket operations.

## Supplemental Information

10.7717/peerj-cs.3040/supp-1 Supplemental Information 1 Raw data.

10.7717/peerj-cs.3040/supp-2 Supplemental Information 2 Code.

10.7717/peerj-cs.3040/supp-3 Supplemental Information 3 Digital Simulation Noise Modeling Setup.

10.7717/peerj-cs.3040/supp-4 Supplemental Information 4 Filter Initial Parameter Configuration.

10.7717/peerj-cs.3040/supp-5 Supplemental Information 5 Filtering parameter Rk performance simulation settings.

10.7717/peerj-cs.3040/supp-6 Supplemental Information 6 Implementation times of different algorithms for a single step run.

10.7717/peerj-cs.3040/supp-7 Supplemental Information 7 Filter Initial Parameter Configuration.

10.7717/peerj-cs.3040/supp-8 Supplemental Information 8 ARMSEs of position velocity and attitude from different algorithms.

## Additional Information and Declarations

### Data Availability

The following information was supplied regarding data availability:

The raw data and code are in the Supplemental Files.
