# Supplementary material for: Adaptive filter parameter reconstruction technology for rocket inertial navigation/satellite integrated navigation system
Source: PeerJ Comput Sci. 2025 Jul 23;11:e3040. doi: 10.7717/peerj-cs.3040 (PMC12453862; doi:10.7717/peerj-cs.3040)
Supplement: Supplemental Information 2 [file peerj-cs-11-3040-s002.zip › Source Code File/Code Program Simulation Determination Description.docx]

Description of the code program simulation

1. the ‘IMU_para_define’ file is a Matlab code file used to implement the reconfiguration of the adaptive filtering parameters, which is placed in the transmitter system during the experimental phase of this thesis, thus implementing the reconfiguration of the airborne navigation system after the parameter estimation is completed.

2. the files within the C program code are code programs for the embedded environment;
