# Supplementary material for: Adaptive filter parameter reconstruction technology for rocket inertial navigation/satellite integrated navigation system
Source: PeerJ Comput Sci. 2025 Jul 23;11:e3040. doi: 10.7717/peerj-cs.3040 (PMC12453862; doi:10.7717/peerj-cs.3040)
Supplement: Supplemental Information 3 [file peerj-cs-11-3040-s003.docx]

Table S1. Digital Simulation Noise Modeling Setup

| Stage | Device | Performance | Parameter |
| --- | --- | --- | --- |
| 1 | Gyroscope | Integrated zero position$(^{\circ}/h)$ $\varepsilon_{x},\varepsilon_{y},\varepsilon_{z}$   | $10,10,10$ |
| 2 | Gyroscope | Angular random walk factor $(^{\circ}/\sqrt{h})$ $N_{ARW_{\omega x}},N_{ARW_{\omega y}},N_{ARW_{\omega z}}$ | $5,5,5$ |
| 3 | Accelerometer | Integrated zero position$(mg)$   | $10,10,10$ |
| 4 | Accelerometer | Angular random walk factor$(mg/\sqrt{Hz})$ $N_{ARW_{Ax}},N_{ARW_{Ay}},N_{ARW_{Az}}$ | $20,20,20$ |
| 5 | GNSS | Velocity measurement noise$(m/s)$ | $0.3,0.3,0.3$ |
| 6 | GNSS | Position measurement noise$(m)$ | $3,3,5$ |
