# Supplementary material for: Adaptive filter parameter reconstruction technology for rocket inertial navigation/satellite integrated navigation system
Source: PeerJ Comput Sci. 2025 Jul 23;11:e3040. doi: 10.7717/peerj-cs.3040 (PMC12453862; doi:10.7717/peerj-cs.3040)
Supplement: Supplemental Information 4 [file peerj-cs-11-3040-s004.docx]

Table S2. Filter Initial Parameter Configuration

| Stage | Filter Parameter | Parametric Value | |
| --- | --- | --- | --- |
| 1 | Initial position $L_{0},\lambda_{0},h_{0}$ | | $[38^{\circ},105^{\circ},900m]$ |
| 2 | Initial velocity $v_{E_{0}},v_{N_{0}},v_{U_{0}}$ | $0_{3\times1}$ | |
| 3 | Initial attitude information  (pitch, yaw, roll) | $[52^{\circ},283^{\circ},0^{\circ}]$ | |
| 4 | Initial state vector $X_{0/0}$ | $0_{23\times1}$ | |
| 5 | Initial associated variance$P_{0/0}$ | $\begin{matrix} diag([(3m)^{2},(3m)^{2},(5m)^{2}, \\ (0.3m/s)^{2},(0.3m/s)^{2},(0.3m/s)^{2}, \\ (0.01rad)^{2},(0.05rad)^{2},(0.01rad)^{2}, \\ (10^{\circ}/h)^{2},(10^{\circ}/h)^{2},(10^{\circ}/h)^{2}, \\ (10mg)^{2},(10mg)^{2},(10mg)^{2}]) \end{matrix}$ | |
| 6 | Initial value of process noise  Covariance $Q_{k}$ | $\begin{matrix} diag([(0.5^{\circ}/\sqrt{h})^{2},(0.5^{\circ}/\sqrt{h})^{2},(0.5^{\circ}/\sqrt{h})^{2}, \\ (3mg/\sqrt{Hz})^{2},(3mg/\sqrt{Hz})^{2},(3mg/\sqrt{Hz})^{2}]) \end{matrix}$ | |
| 7 | Initial value of measurement  noise covariance $R_{k}$ | $\begin{matrix} diag([(3m)^{2},(3m)^{2},(5m)^{2}, \\ (0.3m/s)^{2},(0.3m/s)^{2},(0.3m/s)^{2}]) \end{matrix}$ | |
