# Supplementary material for: Adaptive filter parameter reconstruction technology for rocket inertial navigation/satellite integrated navigation system
Source: PeerJ Comput Sci. 2025 Jul 23;11:e3040. doi: 10.7717/peerj-cs.3040 (PMC12453862; doi:10.7717/peerj-cs.3040)
Supplement: Supplemental Information 5 [file peerj-cs-11-3040-s005.docx]

Table S3. Filtering parameter $R_{k}$ performance simulation settings

| Serial No. | Parametric Value |
| --- | --- |
| 1 | $\begin{matrix} R_{k}=diag([(3m)^{2},(3m)^{2},(5m)^{2}, \\ (0.3m/s)^{2},(0.3m/s)^{2},(0.3m/s)^{2}]) \end{matrix}$ |
| 2 | $0.1R_{k}$ |
| 3 | $0.01R_{k}$ |
| 4 | $10R_{k}$ |
| 5 | $100R_{k}$ |
