# Supplementary material for: Adaptive filter parameter reconstruction technology for rocket inertial navigation/satellite integrated navigation system
Source: PeerJ Comput Sci. 2025 Jul 23;11:e3040. doi: 10.7717/peerj-cs.3040 (PMC12453862; doi:10.7717/peerj-cs.3040)
Supplement: Supplemental Information 6 [file peerj-cs-11-3040-s006.docx]

Table S4. Implementation times of different algorithms for a single step run

| Fliters | Time(ms) |
| --- | --- |
| EKF | 1.41ms |
| IEKF | 2.25ms |
| MVC-EKF | 1.77ms |
| SE2(3)-EKF | 3.19ms |
| AREKF | 1.41ms |
