# Supplementary material for: Adaptive filter parameter reconstruction technology for rocket inertial navigation/satellite integrated navigation system
Source: PeerJ Comput Sci. 2025 Jul 23;11:e3040. doi: 10.7717/peerj-cs.3040 (PMC12453862; doi:10.7717/peerj-cs.3040)
Supplement: Supplemental Information 7 [file peerj-cs-11-3040-s007.docx]

Table S5. Filter Initial Parameter Configuration

| Quantity | Gyroscope | Accelerometer |
| --- | --- | --- |
| Range | $\pm300^{\circ}/s$ | $\pm50g$ |
| Bias | $10^{\circ}/h$ | $10mg$ |
| Random walk | $1^{\circ}/\sqrt{h}$ | $6mg/\sqrt{Hz}$ |
| Update Frequency | $400Hz$ | $400Hz$ |
