# Supplementary material for: Adaptive filter parameter reconstruction technology for rocket inertial navigation/satellite integrated navigation system
Source: PeerJ Comput Sci. 2025 Jul 23;11:e3040. doi: 10.7717/peerj-cs.3040 (PMC12453862; doi:10.7717/peerj-cs.3040)
Supplement: Supplemental Information 8 [file peerj-cs-11-3040-s008.docx]

Table S6. ARMSEs of position velocity and attitude from different algorithms

| Filters | RMSE | | | | | | | | |
| --- | --- | --- | --- | --- | --- | --- | --- | --- | --- |
|  | Lati  (deg) | Longi  (deg) | Alti  (m) | VelN  (m/s) | VelU  (m/s) | VelE  (m/s) | Pitch  (deg) | Yaw  (deg) | Roll  (deg) |
| EKF | 6.67e-6 | 6.07e-6 | 0.965 | 0.549 | 0.578 | 0.527 | 0.159 | 1.641 | 0.245 |
| MVC-EKF | 5.32e-6 | 4.33e-6 | 0.8 | 0.327 | 0.348 | 0.317 | 0.1 | 0.994 | 0.151 |
| IEKF | 5.2e-6 | 3.8e-6 | 0.68 | 0.171 | 0.203 | 0.198 | 0.067 | 0.6 | 0.1 |
| AREKF | 3.52e-6 | 2.78e-6 | 0.49 | 0.111 | 0.118 | 0.109 | 0.032 | 0.329 | 0.048 |
